# Population-Specific Covariation between Immune Function and Color of Nesting Male Threespine Stickleback

**DOI:** 10.1371/journal.pone.0126000

**Published:** 2015-06-03

**Authors:** Daniel I. Bolnick, Kum Chuan Shim, Matthew Schmerer, Chad D. Brock

**Affiliations:** 1 Howard Hughes Medical Institute, University of Texas at Austin, Austin, Texas, United States of America; 2 Department of Integrative Biology, University of Texas at Austin, Austin, Texas, United States of America; CNRS, FRANCE

## Abstract

Multiple biological processes can generate sexual selection on male visual signals such as color. For example, females may prefer colorful males because those males are more readily detected (perceptual bias), or because male color conveys information about male quality and associated direct or indirect benefits to females. For example, male threespine stickleback often exhibit red throat coloration, which females prefer both because red is more visible in certain environments, and red color is correlated with male immune function and parasite load. However, not all light environments favor red nuptial coloration: more tannin-stained water tends to favor the evolution of a melanic male phenotype. Do such population differences in stickleback male color, driven by divergent light environments, lead to changes in the relationship between color and immunity? Here, we show that, within stickleback populations, multiple components of male color (brightness and hue of four body parts) are correlated with multiple immune variables (ROS production, phagocytosis rates, and lymphocyte:leukocyte ratios). Some of these color-immune associations persist across stickleback populations with very different male color patterns, whereas other color-immune associations are population-specific. Overall, lakes with red males exhibit stronger color-immune covariance while melanic male populations exhibit weak if any color-immune associations. Our finding that color-immunity relationships are labile implies that any evolution of male color traits (e.g., due to female perceptual bias in a given light environment), can alter the utility of color as an indicator of male quality.

## Introduction

Animal mating signals are among the most impressive facets of biological diversity. Consequently, biologists have proposed a variety of models of sexual selection to explain signal evolution [1–3], including Fisherian runaway sexual selection [4,5], perception bias by the choosy sex [6,7], intrasexual competition [8], and various handicap models in which signals convey information about the signaler’s likely quality as a mate [9,10]. Despite extensive effort devoted to testing these models of sexual selection, they remain difficult to disentangle [2,7], in part because the various models are not mutually exclusive [11].

Multiple forms of sexual selection can act concurrently on a single trait. For example, bright male colors can serve to attract female attention, by making the male more visible against background light [12–14]. As a result, in many species, sexual selection for greater visibility to females drives divergent evolution of male color between populations inhabiting different light environments [15–21]. However, females’ perceptual bias may not be the only form of sexual selection acting on male color. Many studies suggest that females also prefer colorful males because this color conveys some aspect of male quality [22,23], and thus the potential for direct or indirect benefits to the female [24]. In many species male color is correlated with immunocompetence or parasite load [22,25–30], and females prefer males whose signals coincide with low parasite load or robust immunity [12].

Because multiple forms of sexual selection can concurrently act on male color, there is the potential for interactions between these forces. Specifically, evolution of male color in response to female perceptual biases may alter the relationship between color and immune traits, thereby undermining the potential for females to choose based on male quality. For example, Wong et al. [21] showed that stickleback males’ sexual displays were an effective signal of male condition in clear water, but anthropogenic algal blooms both reduced male redness and made this signal harder to see. When such environment-dependent signal transmission drives evolution of male color, it may also alter the relationship between color and immunity. Consequently, we predicted that populations inhabiting different light environments would exhibit different relationships between male color and immunity. Surprisingly, little is known about the repeatability of male color-immune correlations across populations, despite many previous studies demonstrating color-immunity correlations within single populations[22,25–30], and many studies of between-population differences in male color [15–21]. We tested whether male color and immune traits covary in natural populations of threespine stickleback (*Gasterosteus aculeatus*), and whether the color-immune relationship is consistent across populations in different light environments.

### Study system

In most populations throughout sticklebacks’ circumpolar range, breeding males exhibit a red throat, a translucent or dark back, and iridescent blue eye. The iconic red throat has been the subject of extensive research, showing that females generally prefer red males [12,31–36]. Variation in color has heritable and environmental components [37,38]. Environment effects exist because fish are unable to synthesize the red pigments *de novo*; these pigments are produced in part using carotenoids obtained from food. Also, stress (including but not limited to male-male conflict) can cause individuals to dynamically modulate their color.

Multiple mechanisms appear to underlie female sticklebacks’ preference for males with redder throats. Visual modeling of light spectrum reflectance, water transmission, and female visual reception shows that the red throat increases male visibility against background light in certain light environments [38–41]. Further support for this female perceptual bias model comes from well-established correlations between males’ color and their light environment [19,39–41]. Red-chinned stickleback tend to be found in clearer water, where red increases their visibility. In tannin-stained habitats where the signaling environment is shifted towards longer wavelengths [15,39], red chins provide poor contrast and stickleback instead evolve a dark black or iridescent blue ‘melanic’ phenotype which provides greater contrast with the background. In the present study we use this background knowledge to justify our choice of four stickleback populations, two from clearer lakes with red-chinned males and two from more tannic lakes with melanic males.

There is also evidence that female stickleback prefer redder males because brighter red pigments indicate higher quality mates [12,22,33]. Redder male stickleback are in better condition [42,43], better fed [44], and carry fewer parasites [12, 31, 45–47] (but see [48]), and have more parasite-resistant offspring in laboratory trials [24]. Despite this positive association, other studies have found that redder males are immunocompromised. Experimentally elevated 11-ketotestosterone induced redder throats but suppressed innate immune function including ROS [49,50]. This trade off occurs because the carotenoids required to generate red throat pigments are also valuable anti-oxidants which protect host tissues against oxidative stress during immune responses involving reactive oxygen species (ROS) [26,32,51–55]. Consequently, it is widely believed that when dietary carotenoids are scarce, males face a trade-off between devoting carotenoids to nuptial coloration or protection against immunopathology.

We are thus left with apparently contradictory conclusions that red coloration compromises some measures of immune function, but nevertheless indicates low parasite load and higher resistance. This contradiction may reflect the complex relationship between parasite resistance and the few available measures of stickleback cellular immune function. However it is also possible that contradictory results reflect genuine among-population differences in color-immune relationships [34]. Such population differences are to be expected when, as described above, different environments lead to the evolution of different male color traits. Color signals of male quality are of no value when the color cannot be effectively reflected or transmitted through the environment. Also, trade-offs between using carotenoids for red coloration versus immune function are rendered moot when males do not express a red throat. Thus, the experimentally demonstrated connection between red coloration and immune function [49, 50] may be overridden when female perceptual biases favor melanic (non-red) males in more turbid habitats.

We therefore hypothesized that stickleback populations inhabiting different signaling environments exhibit different color-immunity relationships. Specifically, we predicted that relationships between immune function and male color will differ between mostly red and mostly melanic populations. To test this prediction, we measured color-immunity relationships in each of four populations of stickleback from lakes in northern Vancouver Island, British Columbia. Because we used wild-caught animals to capture natural variation in color and immunity, these relationships are strictly correlational. However, it is exactly this natural correlation, with all its confounding variables and sources of noise, which would be most relevant to female mate choice decisions.

## Methods

### Collection

**Fig 1:**
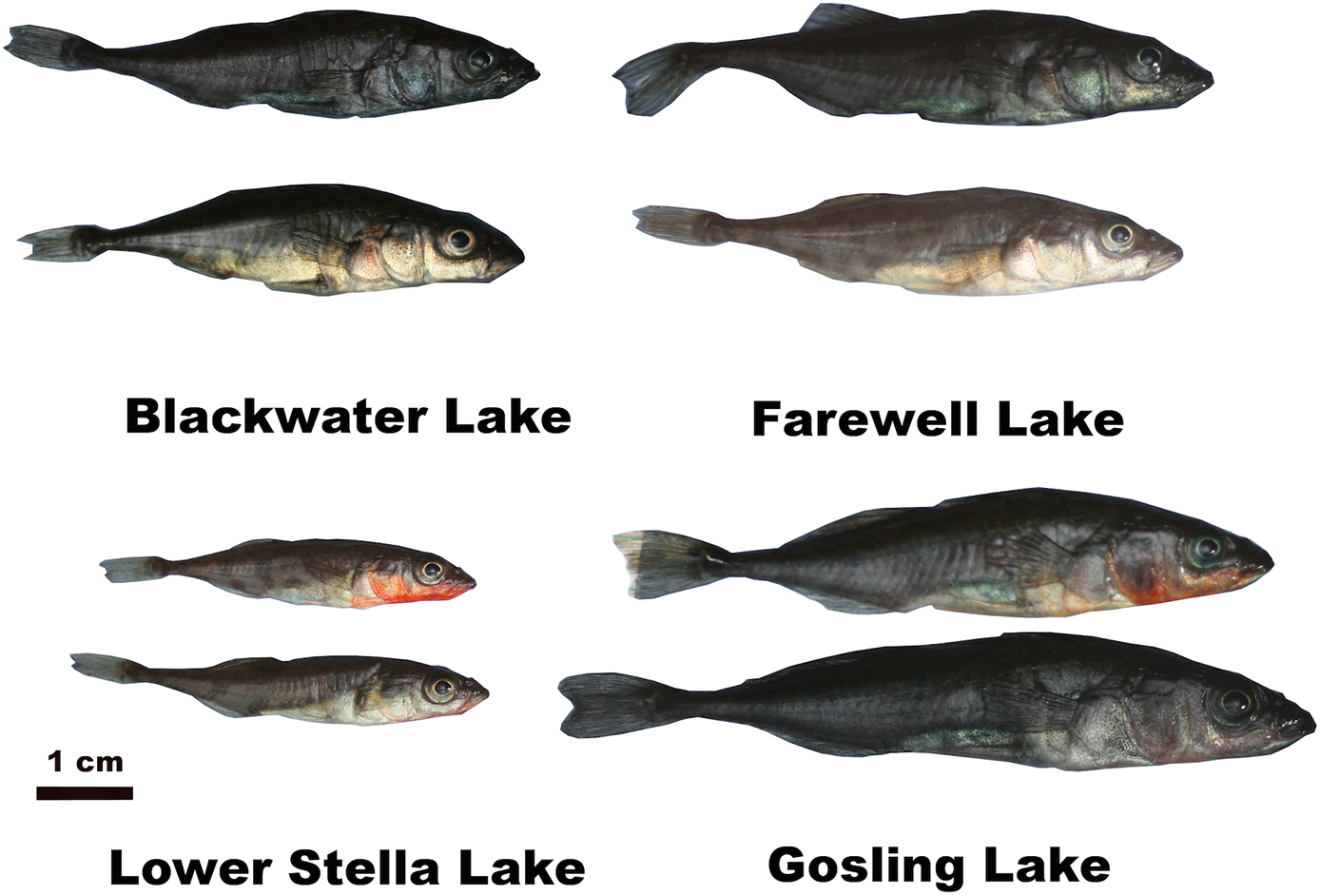
Variation in male color within and among populations of threespine stickleback studied in this paper. Two males are shown per lake to illustrate the range of color within populations, as well as between-population differences. All males shown here were actively nesting. Note that the very melanic male from Gosling Lake (bottom right) is exceptional for that population, most individuals from that lake resemble the red-chinned individual. Photographs were taken under standardized lighting conditions.

During the breeding season (May-June 2011), we collected ≈40 nesting male stickleback from each of four lakes on Vancouver Island (Gosling, Lower Stella, Blackwater, and Farewell Lakes). The light environment differs between these lakes, in terms of both total irradiance and the relative composition of short and long wavelengths (S1 Fig). As expected from prior studies [39], male color differs between these divergent light environments. In clearer lakes (Gosling and Lower Stella) males exhibit the classic red-throated nuptial coloration, whereas in turbid lakes (Blackwater and Farewell) males are iridescent blue/black. However, there is appreciable variation within each lake (Fig 1). These sites represent contrasting ends of the among-lake variation in light environment and stickleback color in this region.

A snorkeler (CDB) searched for nesting or territorial males along the shoreline of each lake (nests were 0.25–2.5 meters deep). The snorkeler observed each male for 10 minutes to confirm nesting status, presence/absence of eggs or fry, and male behavior (aggression, courting). The male was then caught by dipnet. Collections were conducted with permission of the British Columbia Ministry of Forest, Lands and Natural Resource Operations. All collections and methods used in this study were approved by the University of Texas at Austin Institutional Animal Care and Use Committee (IACUC; Protocol AUP-2011-00044). Specimens were collected from public land, and did not entail collection of protected species. Details of collection location and sample sizes are provided in S1 Table.

Following capture, males were immediately placed alone in a black mesh container at 1m depth in the lake, to minimize subsequent color changes that might be induced by male-male interactions or shifts in the light environment. Males were held in the dark until enough males were captured for the day, at which point we measured all males’ color and immune traits. Typically, males were held for between 1.5 to 3.5 hours. Each male was removed from the lake in turn, and immediately anaesthetized (within ~ 1 minute) and measured for color, then immediately euthanized and head kidneys removed for immune measurements described below. Immune measurements were carried out in a mobile immunology laboratory (a modified RV trailer) parked at the lakes to minimize travel time. Thus, we designed our study to minimize, as much as possible, handling time effects on both male color and immunity.

### Color measurement

Males were lightly anaesthetized in MS-222, and their color (reflectance spectra) was measured in triplicate at four body locations (lower eye, lower jaw, preoperculum, and abdomen), using an EPP200C UV-VIS spectrophotometer, SL-4 Xenon lamp, and R400-7 reflectance probe at a 3-mm distance from the fish (Stellarnet, Tampa, FL). Spectralon white standard measurements were taken between fish to account for amp drift. For each measurement, we binned reflectance data into 10 nm intervals from 300 to 700 nm, then averaged reflectance within each bin and across triplicate readings, to yield mean reflectance for each wavelength bin. Summing reflectance across all bins quantifies overall brightness, whereas the proportion of brightness in each bin gives a measure of color. Here, we focus on brightness and the proportion of red reflectance (571 to 700 nm as a proportion of total reflectance). Spectrophotometric data from Farewell Lake was corrupted by hardware failure, so we analyze color data from only three lakes, but immune data from four lakes. To compensate for the loss of data from this one lake, we also measured color from photographs of all fish. Methodological details of photographic color data, and results of color-immune associations, are presented in S2 File.

### Immune function measurements

Here we give a brief overview of immune measurement methods, which are modified from [56–60]. See S1 File for detailed protocol description. Within one minute after color measurements, each male was euthanized, weighed, and we dissected out the head kidneys (the major hematopoietic organ in fish), which we used to measure three immune traits: (i) the relative abundance of granulocytes and lymphocytes, (ii) ROS production, and (iii) phagocytosis rates. The granulocyte/lymphocyte ratio is indicative of individuals’ relative investment in innate/adaptive immune cell populations. Reactive oxygen species (ROS) include a variety of oxidizing compounds secreted by phagocytic cells, which can cause oxidative damage to the surface of parasites (and host tissues). Phagocytposis rates measure the proportion of granulocytes that engulf foreign particles in a fixed time. The process of phagocytosis is involved in both direct degradation of pathogen particles, as well as acquiring pathogen antigens which MHC then presents to adaptive immune cells. Note that the fitness value of these traits can be context-dependent, so we do not consider these as measures of “good” immune function. Rather, we view these traits as axes of natural variation in immune strategy, without presupposing particular value.

Head kidneys were held in culture medium in individual 0.5 ml centrifuge tubes on ice for up to 2.25 hours while the remaining head kidneys were acquired from all males captured that day. Holding time had no significant effect on subsequent immune measurements. We then created a single-cell suspension of each sample (see S2 File for details) and used the cell suspension to measure the following immune traits in triplicate.

First, we ran a cell suspension (with propidium iodide [PI] to identify dead cells) through an Accuri C6 flow cytometer to coarsely classify and count live cells, collecting data on 50,000 particles. These particles include cellular debris (too small to be cells) and dead cells (PI^+^), which were subtracted from the dataset. Live cells were classified as either as lymphocytes or granulocytes based on their combination of cell size (forward scatter) and internal complexity (side scatter). We calculated the total number of live cells sampled, and the proportion of granulocytes and lymphocytes. Note that these cell types are coarse categories, each of which includes heterogeneous populations of cell types that cannot presently be distinguished in stickleback.

We incubated aliquots of the cell suspension with Dihydrorhodamine 123 (DHR), which fluoresces green when exposed to oxygen radicals. The intensity of green flourescence per cell reflects the magnitude of ROS production by either unstimulated cells, or in cells stimulated by Phorbol myristate acetate (PMA) [60]. ROS production is measured as the difference in median DHR fluorescence between control (DHR only) and stimulated (DHR + PMA) suspensions, averaged across triplicates. Unlike prior approaches to measuring ROS in stickleback, which depended on multi-day culture and bulk chemiluminescence [56–60], our rapid ROS protocol mitigates changes in cell viability and ratios during extended culture, and allows us to focus on ROS production by granulocytes specifically. After gating on granulocytes (to remove other cells from consideration), we calculated the induced ROS production as the difference between DHR^+^/PMA^+^ versus DHR^+^ cultures. Thus our measure of ROS production represents the increase in ROS within PMA-stimulated granulocytes, above background fluorescence.

Finally, we incubated an aliquot of cells overnight with or without fluorescent beads at 18°C for 18 hours, then used flow cytometry detection of bead fluorescence to count granulocytes and lymphocytes with and without beads. We used forward- and side-scatter gating to focus on the fraction of granulocytes with beads, as lymphocytes phagocytize at a lower (but non-zero) rate [61].

### Analyses: Do populations differ in immune function or color?

We tested whether populations differ in immune function or color. We focused on three metrics of immune function: the proportion of granulocytes, ROS production by granulocytes, and the proportion of phagocytic granulocytes at 18°C. We then compared these three immunological metrics to spectrophotometric measures of male brightness and hue on each of four body parts (eye, throat, abdomen, preoperculum). Because spectrophotometric color data were corrupted from one lake, we also compared immune traits to photographic measures of color. Because these photographic data yielded comparable results to the more precise spectrophotometric data, we focus on the latter here, and relegate the photographic data to S2 File. We used MANOVAs to test whether immune function differs significantly among populations, using individuals as the level of replication (averaging triplicate trait measures). Likewise, we used MANOVAs to test for among-population differences in male color, with a dependent matrix including total brightness, and relative reflectance in the UV-blue, yellow-green, and orange-red bins. All statistical analyses were carried out in R [62], after testing model assumptions.

### Analyses: Are color and immune traits correlated within populations?

We used canonical correlation analyses (CCA) to test for multivariate correlations between male color (total brightness and relative red reflectance of each body part) and immune phenotypes (proportion of granulocytes, ROS production, and granulocyte phagocytosis rates). We used an asymptotic test of significance (R package CCP) to evaluate the null hypothesis that color and immunity are unrelated. We performed the CCA for each population separately. We also used CCA on all 4 lakes together, after mean-standardizing each trait within each population to avoid correlations due to between-population trait differences. The CCA loadings indicate the relative contribution of particular color and immune traits towards such correlations. Comparable CCA results for photographic color measures are detailed in S2 File.

We then statistically tested the role of particular combinations of color and immune traits, in generating the observed multivariate canonical correlations. Within each lake, we used linear regression to evaluate the associations between single immune traits (dependent variables: proportion of granulocytes, or ROS production, or phagocytosis rates), and single color traits (brightness or relative red reflectance of each of the four body parts). Because the CCA analyses demonstrated that immune-color correlations do exist, we do not apply P-value adjustments for multiple comparisons. Instead, we treat these numerous regressions as a way to identify the univariate trait combinations driving already-confirmed canonical correlations, rather than *de novo* hypothesis tests.

A key limitation of CCA is that it is not effective at detecting group-specific (e.g., lake-specific) canonical axes. Therefore, to test whether color-immune relationships are parallel across lakes, or differ among lakes, we used all samples in an ANCOVA testing whether a given immune trait depends on lake, a main effect of color (indicating a shared color-immunity association), or a lake by color interaction (indicating lake-specific color-immune relationships). Again, because our CCA analyses had already identified significant color-immune relationships in some lakes but not others (see results), we treat these ANCOVAs as a descriptive tool and do not apply P-value adjustments. Lake was treated as a fixed effect because lakes were selected deliberately.

Color-immunity relationships could be indirect results of their joint correlation with some other covariate. To account for such potentially spurious color-immune associations, we considered many possible confounding variables (see S3 File for details). These covariates include infection intensity by various macroparasites, overall parasite load, fish size, breeding status (guarding offspring or not; courting or not), collection date and time, and handling time between capture and measurement. Because we consider so many possible covariates, we lacked the power to include all covariates within every color-immune linear model. Instead, we identified any covariates which were significantly associated with both a color and an immune trait (using a liberal cut-off for significance, P < 0.1), and included those most-plausible covariates in the linear models of immunity as a function of color. Detailed methods and results are relegated to S3 File, because none of these covariates appreciably affected the color-immunity correlations described below.

## Results

### Immune function varies within and among populations

We found significant among-population variation in all measured immunological traits (MANOVA, P < 0.0001), including the relative abundance of cell types, ROS intensity, and phagocytosis rates of granulocytes (S2 Fig). We also found substantial among-individual variation in immune function within each of the four populations, using triplicate immune measures on each individual to calculate residual error (individual effect [nested within lake] F > 5 and P < 0.0001 for all dependent variables). An important caveat is that the among-individual variances are based on technical replicate measurements from a single culture of head kidney cells, rather than repeated sampling of individuals at multiple time points.

As another measure of repeatability and among-individual variation, we calculated the correlation between duplicate measures, mean-standardizing by lake to remove effects of among-lake variation and randomly picking two of the triplicate measures per fish. The correlations between duplicate measures of cell counts, proportion of granulocytes, ROS production, and phagocytosis rates were r = 0.95, 0.74, 0.83, and 0.90 respectively (all P < 0.0001). Among-individual variation in immune function is not driven simply by body size variation. With the exception of total cell count (which depends on the size of head kidneys used to generate cell cultures), none of the other immune measures were significantly correlated with fish mass (S3 File).

### Color varies within and among populations

Male nuptial color varies significantly both within and between populations (Fig 1). Brightness differed between lakes for the lower eye (F_2,119_ = 3.1, P = 0.047), preoperculum (F_2,119_ = 18.3, P < 0.001), and abdomen (F_2,119_ = 7.2, P = 0.001) but not throat (F_2,119_ = 0.35, P = 0.705). Color (normalized to unit brightness) differed significantly between lakes for all four body parts (S3 Fig; P < 0.001 for all variables except UV-blue reflectance of the preoperculum, P = 0.598). Gosling Lake males have the bluest eyes, Lower Stella males have the reddest preoperculum and throats, while Blackwater Lake males have the bluest throats. Abdomen color is less variable, but tends to be bluer in Gosling Lake. Finally, nested ANOVAs using replicate measures for residual errors, confirmed that there is significant among-individual variation in color within populations, for total brightness and the proportion red reflectance of all traits (all P < 0.001). Photographic measures of color also varied within and among lakes, and were typically moderately correlated with spectrophotometric measures (S2 File).

### Color and immune traits are correlated within populations

Within populations, immune function is correlated with male color. We observed significant canonical correlations between color (8 variables) and immunity (3 variables) within each of the two red-male lakes (Gosling and Lower Stella, both P < 0.004) but not the one melanic population for which we had spectrophotometric data (Blackwater, P = 0.424). These Gosling and Lower Stella canonical correlations remain significant after Bonferroni correction for multiple comparisons. The canonical axis loadings indicate which traits drive these multivariate correlations (Table 1). In Gosling and Lower Stella lakes, male hue (red reflectance), rather than total brightness, is the most relevant color trait. Granulocyte relative abundance and phagocytosis rates explain most of the immunological axis that covaries with hue, whereas ROS contributes little to the covariance.

**Table 1:** Canonical correlation analysis (CCA) of the multivariate correlation between three immune traits and multiple dimensions of male color measured via spectrophotometry.

	All 4 lakes	Blackwater	Gosling	Lower Stella
**Test statistics**				
r	0.540	0.484	0.745	0.670
P	**0.0003**	0.4240	**0.0010**	**0.0034**
**Canonial X axis loadings**				
Brightness				
Abdomen	0	0	0	0
Lower eye	0	0	0	0
Preoperculum	0	0	0	0
Throat	0	0	0	0
Proportion red reflectance				
Abdomen	-1.66	1.14	-2.00	1.93
Lower eye	-0.62	1.56	-1.63	1.52
Preoperculum	0.93	-2.93	1.23	-0.42
Throat	0.46	-4.73	0.16	-2.31
**Canonical Y axis loadings**				
% granulocytes	-0.65	1.22	-0.58	1.48
Median ROS production	0.028	0.24	-0.01	-0.07
% phagocytic granulocytes	-0.469	-0.03	-0.73	-0.471

CCA was run first for all four populations combined, after first mean-standardizing each trait within each population, to avoid correlations arising from between-population differences. CCA was then run separately for each lake (except Farewell, due to data corruption). For each CCA, we present the correlation coefficient (r), and an asymptotic approximation to an F statistic and P-value, obtained via the p.asym command in the R package CCP. We also present the loadings of each trait on the canonical x axis (for color) and y axis (for immune traits).

Although hue was significantly associated with granulocyte number and activity in both lakes, the particular effects differed between populations. In Gosling Lake, both phagocytosis and (to a lesser extent) granulocyte abundance tended to be higher in males with redder abdomens and eyes, and less red preopercula (Table 1). In contrast, in Lower Stella phagocytosis rates increased but granulocyte abundance decreased in redder males, and throat color had the strongest effect. In both lakes, redder abdomens and lower eyes coincided with more granulocytes. In contrast, phagocytosis rates responded in opposite directions between lakes; redder abdomens indicate higher phagocytosis rates in Gosling, but lower phagocytosis rates in Lower Stella. Despite these lake-specific trends, associations were sufficiently similar across lakes that a single CCA on all lakes together found significant color-immune associations (P = 0.0003). In particular, males with redder abdomens tended to have relative more granulocytes, a trend seen within each lake separately (including Blackwater, though it was not significant). Photographic measures of color yield comparable CCA results (S2 File). Namely, (i) photograph color and immunity are correlated in the two red-male lakes but not the two melanic-male lakes, (ii) hue rather than brightness explains most immunological variation, (iii) ROS contributes little to the canonical correlation, and (iv) specific color and immune trait combinations can differ among populations.

We used linear models to investigate particular combinations of color and immune traits, and to test for lake-specific effects (e.g., immunity depending on a lake*color interaction). The results of these linear models are summarized in S4 Fig and S3 Table. Linear models based on photographic measures of color are presented in S2 File, and broadly confirm the following results from spectrophotometric data.

**Fig 2:**
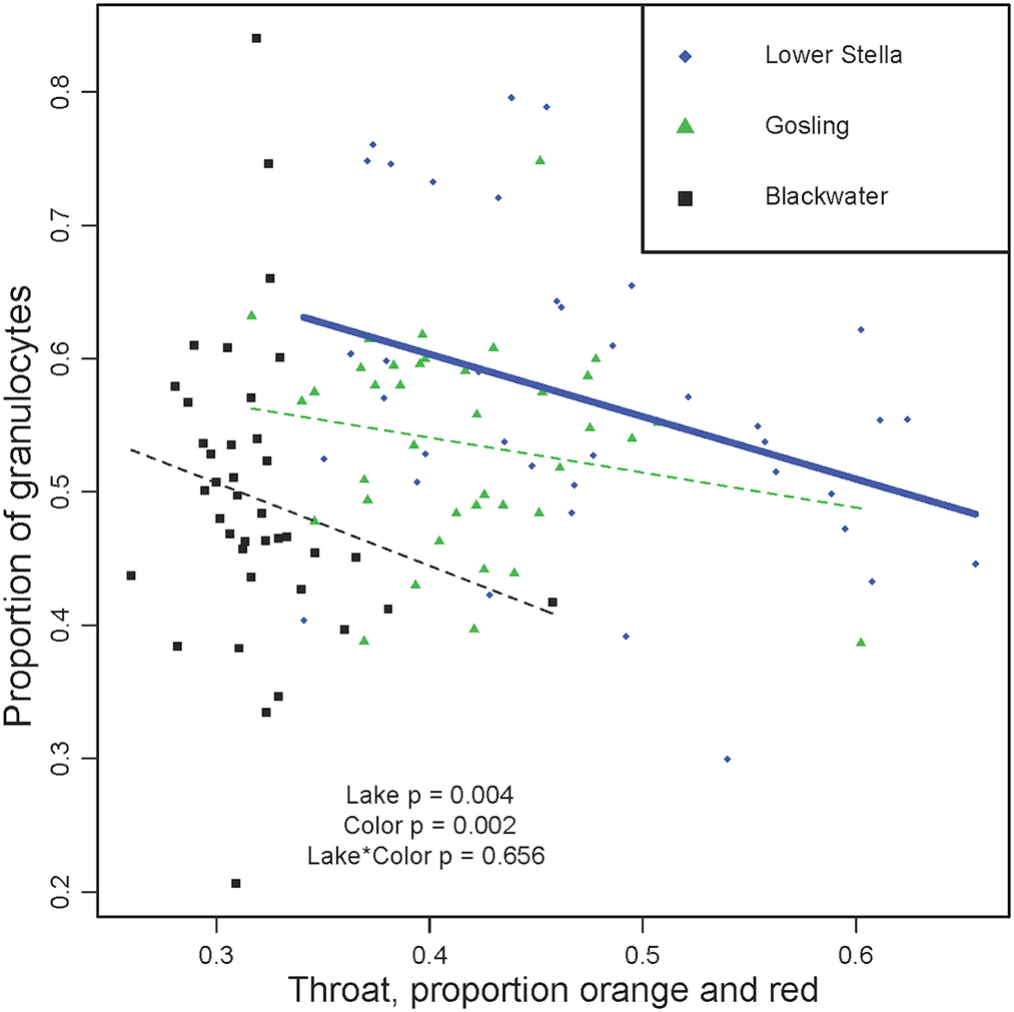
Male color (proportion red reflectance of throat) is negatively correlated with the proportion of granulocytes within populations. While the trend is only significant within Lower Stella (r = 0.353, P = 0.009), the slope is very similar in the other two lakes (Blackwater, r = -0.181; Gosling, r = -0.185) leading to an overall effect of color (P = 0.002) and no color*lake interaction. Symbols and colors denote populations (see legend). A separate trendline is drawn for each population, using a thick solid line to denote a significant correlation, or thin dashed line when non-significant.

Consistent with the CCA loadings, linear regression confirmed that males with redder throats have fewer granulocytes (Fig 2; ANOVA effect of color F_1,109_ = 9.6, P = 0.002, lake F_2,109_ = 5.7 P = 0.004). Notably, for this trait there is no significant lake*color interaction (F_2,109_ = 0.422 P = 0.656), demonstrating that despite the vast difference in overall throat redness among lakes, there is no detectable lake-to-lake difference in the association between throat redness and granulocyte abundance. This result is consistent with previously described trade-offs between red signaling and innate immunity [49]. Although CCA identified an even stronger effect of abdomen color on granulocyte frequency, in univariate analyses the other body parts had no significant effects (eye, preoperculum, abdomen; all P> 0.1). No parasitological, reproductive, or methodological covariates were significantly associated with granulocyte frequency (S3 File), and so no other covariates were included in these linear models. Photographic color also supports a negative relationship between red (intensity and area) and granulocyte abundance.

Consistent with the CCA results, ROS production showed few associations with male color. Across all lakes, ROS tends to be higher in fish with bluer eyes (color P = 0.017, no color*lake interaction), although within individual lakes this trend is only significant in Blackwater Lake (P = 0.015). This regression includes, as a covariate, infection intensity by the parasitic copepod *Thersitina*, which is also correlated with both eye color and ROS production. Including or excluding this parasite from the the linear model does not appreciably change the relationship between eye color and ROS. There is also a weakly significant tendency for ROS to be lower in males with redder abdomens (color P = 0.048, no color*lake interaction, and a significant lake*Thersitina interaction P = 0.012), although this trend is not significant within any individual lake.

**Fig 3:**
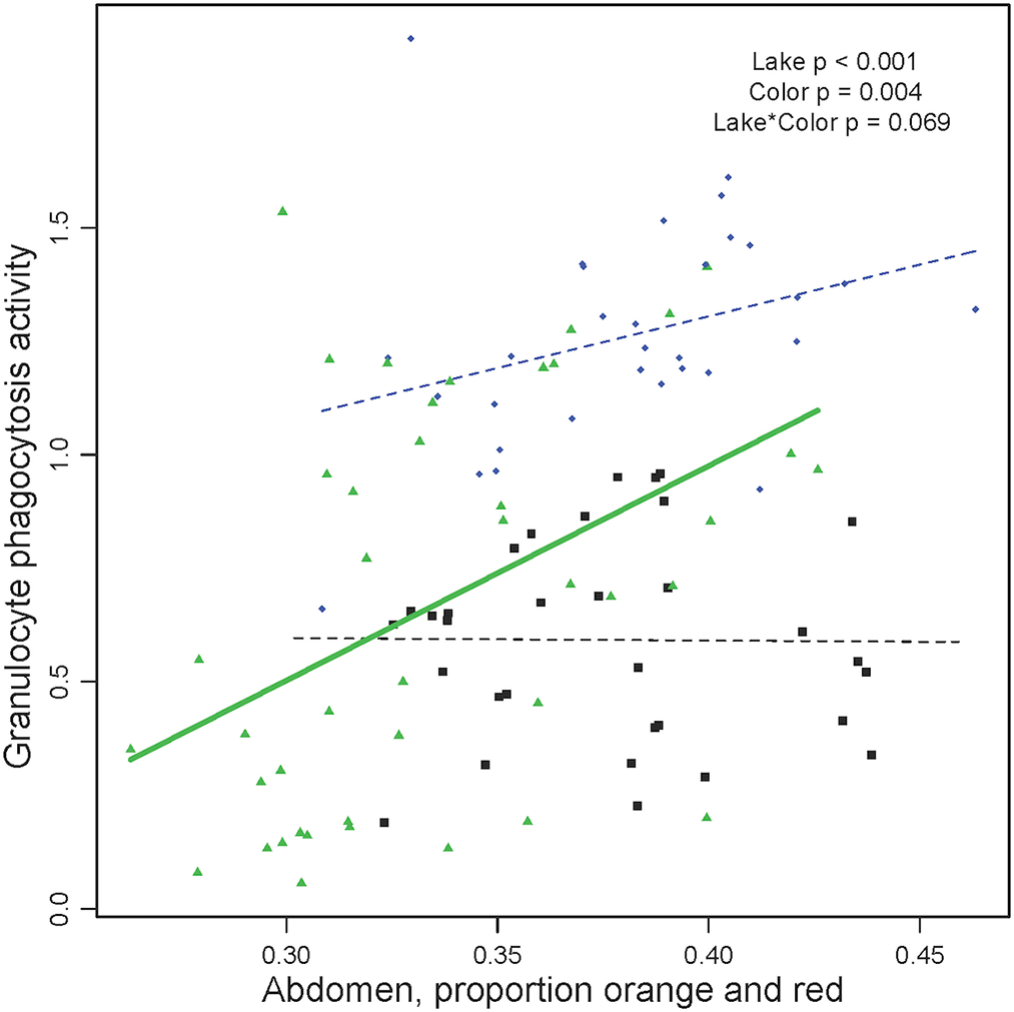
Male color (proportion red reflectance of abdomen) exhibits lake-specific correlations with the phagocytosis rate. The trend is significant within Gosling Lake (r = 0.438) and in a similar direction in Lower Stella Lake (r = 0.327) but not Blackwater Lake (r = -0.009) leading to a marginally significant lake*color interaction. Symbols and colors denote populations, as in Fig 2. A separate trendline is drawn for each population, using a thick solid line to denote a significant correlation, or thin dashed line when non-significant.

Granulocyte phagocytosis activity was higher in males with redder abdomens (Fig 3). This trend was most pronounced in Gosling Lake (P = 0.010), but was in the same direction in Lower Stella (P = 0.112) leading to significant main effect of color (ANOVA lake F_2,97_ = 38.9, P < 0.0001, color F_1,97_ = 6.7, P = 0.011). However, this trend was absent within Blackwater Lake (P = 0.963), generating a marginally significant lake*color interaction in the full 3-lake model (F_2,97_ = 2.63, P = 0.077). This linear model included collection date (and a date*lake interaction) as a covariate, because date was the one potentially confounding variable we examined which was significantly correlated with both phagocytosis rate and abdomen color. Other body parts do not show an association between color and phagocytosis (a full summary of lake, color, and lake*color interaction effects, for each body part and each immune trait is presented in S2 Table and S3 Table). Regression of phagocytosis on preoperculum color included handling time and relative parasite load as covariates. Regression of phagocytosis on throat color included collection date and time as covariates. Phagocytosis rates were also correlated with photographic measures of male color (S2 File).

**Fig 4:**
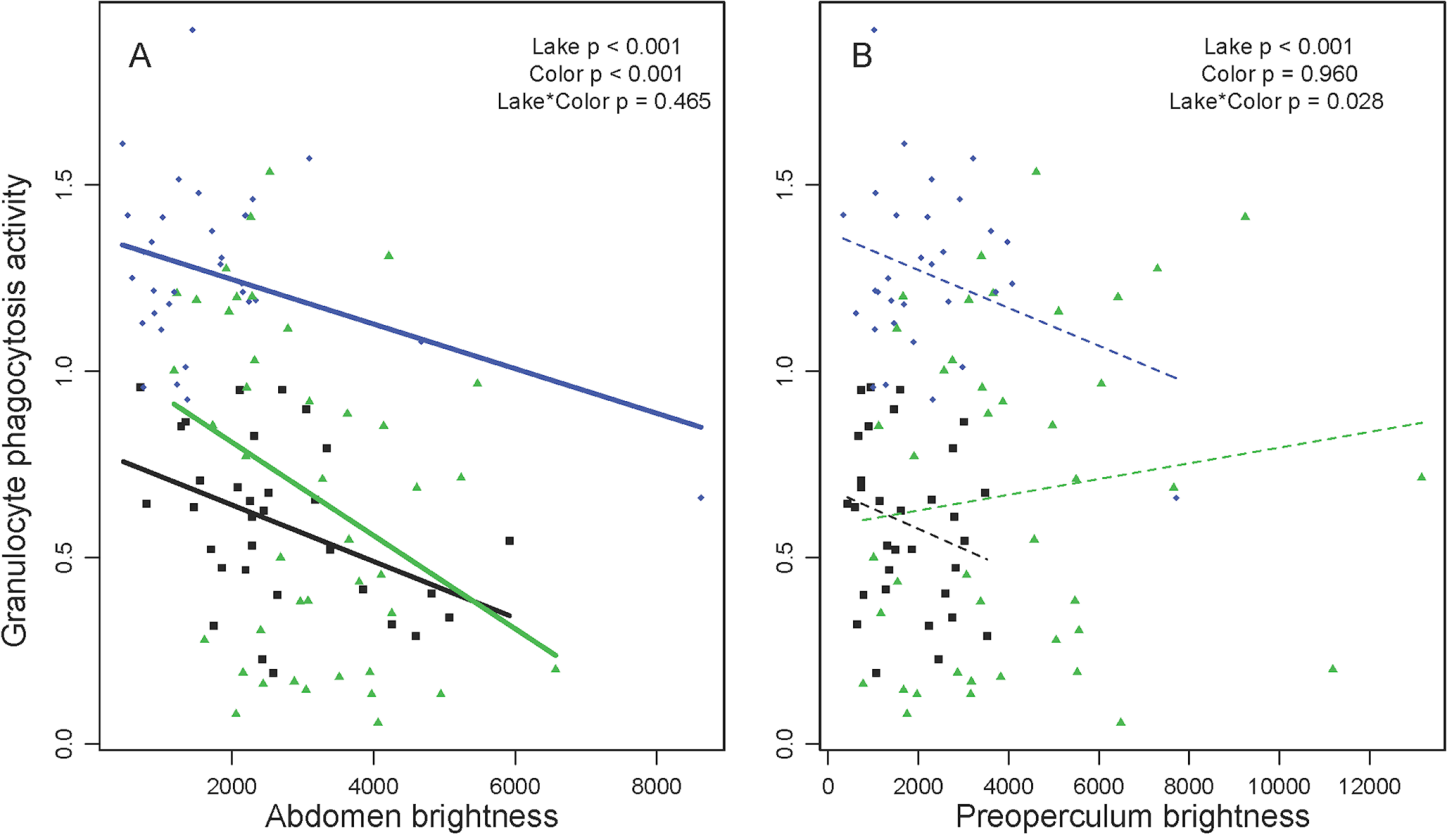
Granulocyte phagocytosis rates are negatively correlated with male brightness (total reflectance). (A) For abdomen brightness r = -0.426, -0.347, -0.386 respectively for Blackwater, Gosling, and Lower Stella Lakes. (B) For preoperculum brightness, r = -0.231, 0.129, and -0.304 respectively. Symbols and colors denote populations, as in Fig 2. The results from an ANCOVA are listed within each panel. A separate trendline is drawn for each population, using a thick solid line to denote a significant correlation (P < 0.05), or thin dashed line when non-significant. Throat and eye brightness effects are not presented here, for brevity, but closely resemble (A).

Although the CCA did not give any weight to male brightness, univariate linear regressions suggest that there are associations between brightness of individual body parts and phagocytosis rates. For all four body parts examined, brighter males tend to have lower phagocytosis rates by granulocytes (Fig 4). For both lower eye brightness and throat brightness, there is a significant main effect of color (both P < 0.05) across all four lakes. Testing each lake separately, these trends are only significant within Lower Stella Lake. Brighter abdomens also coincide with lower phagocytosis rates, overall and within each of the three lakes separately (Fig 4A), with no significant differences in effect slope. Thus, the association between male brightness and phagocytosis rate is generally consistent across populations. The one exception is for preoperculum brightness, which is also negatively correlated with phagocytosis in Lower Stella, but not Gosling, leading to a significant color by lake interaction (P = 0.028, Fig 4B). Such interactions are noteworthy because they support the hypothesis that color-immune relationships vary among populations. No parasitological or other covariates were associated with both phagocytosis and male brightness, so no additional covariates were included in these models.

The linear model results (supporting an effect of male brightness) disagreed with the CCA, which gave brightness negligible weight. This could occur if hue has a relatively stronger effect, so that brightness is relegated to higher-order x axes. We therefore repeated CCAs using only brightness of the four body parts, excluding male hue data. These new CCAs confirmed a general effect of brightness (across all three lakes, r = 0.421, P = 0.035). In lake-specific CCAs, brightness was significantly associated with immunity in Gosling (P = 0.028), Lower Stella (P = 0.021), but not Blackwater (P = 0.467). In each lake, and in the combined CCA, phagocytosis rates were by far the most strongly loaded immune trait driving these multivariate correlations.

In an observational correlative study such as this, correlations between traits could arise from those traits’ joint association with some other variable. S3 File presents an extensive analysis of multiple potentially confounding variables. Some of these variables, such as parasite load, are not so much confounding effects as they are potential mechanisms. Variation in parasite resistance among individuals, with corresponding differences in immune response and condition-dependent color, could be the reason why color and immunity are correlated. As detailed in S3 File and illustrated in S5 Fig, we did observe numerous associations between parasite load and color or immunity. Infection intensities of several parasite taxa were correlated with male brightness and/or hue. For instance, in Gosling Lake only, males with higher *Schistocephalus solidus* loads tended to be brighter. In Lower Stella only, brighter males had lower Unionidae infection loads. Likewise, effect directions varied for parasite-hue associations (S3 File). Fewer associations were observed with immune variables, although Thersitina and Proteocephalus loads parasites were both positively correlated with ROS production. Some non-parasitological variables were also correlated with male color (mostly hue) and immunity (phagocytosis rates), summarized in S5 Fig and S3 File. Male courtship state, capture date and capture time, and post-capture handling time were all correlated with color and/or phagocytosis rate. In all cases where a parasitological or other covariate were correlated both a color and an immune trait, including the covariate(s) in the immune—color linear regression had no qualitative effect on our inferences.

## Discussion

Prior research has clearly established that populations inhabiting different light environments are subject to divergent sexual selection on color signals [39,63,64]. In a variety of organisms including stickleback, these same color signals have previously been shown to covary with male immune phenotype or parasite load. Male color evolution, in response to local light environments, might therefore have the side effect of modifying the relationship between color and immunity. However, there is little data regarding the repeatability (or lack thereof) of color-immune relationships. We addressed this gap by evaluating whether color-immune correlations occur in wild threespine stickleback (as has previously been found in laboratory stickleback [49]), and whether these correlations are conserved across populations or are population-specific. Specifically, we examined populations that we knew, in advance, inhabited different light environments and exhibited corresponding differences in male color, consistent with previous studies of male color evolution in stickleback [38–41]. Males are predominantly red in the two lakes with blue-shifted wavelengths and brighter irradiance, whereas males are predominantly blue-black in the two lakes with lower red-shifted irradiance. Importantly, we also observed substantial within-population variation in both male color (brightness and hue) and immune phenotypes.

Consistent with prior studies of male color and condition in stickleback [12,24,31, 42, 43, 49, 50], we found significant associations between male color and immune function. However, most of the specific associations we found have not been described before. For example, in all populations examined, males with brighter abdomens have granulocytes with lower phagocytic activity, and in one population (Lower Stella) phagocytosis was negatively correlated with brightness of all body parts. Phagocytosis is an essential immune function that can lead to direct elimination of pathogens by both granulocytes and B-cells [61], by engulfing small pathogens or killing infected host cells. This is an essential component of innate immune clearance of pathogens. Phagocytosis is also required to initiate adaptive immune response as antigen presenting cells must phagocytose pathogen antigens in order to present them via MHC class II and activate CD4+ T cells. Previous studies have not found associations between brightness and phagocytosis, and the mechanism underlying this effect is not known. Our data clearly show that brighter males have less pronounced phagocytosis activity, suggesting reduced capacity to mount both innate and adaptive immune responses. Importantly, the relationship between phagocytosis and preoperculum brightness differed among populations, indicating that this association is labile.

Controlling for brightness, hue is also associated with immunity. In fact, canonical correlation analyses indicates that hue is the primary covariate of immune traits. Overall, we find that males with redder throats have proportionally fewer granulocytes, suggesting reduced capacity for innate immune responses by primary phagocytic cells. This is consistent with the previously demonstrated trade-offs between innate immune function and male red coloration [49]. However, unlike the experimental lab study of endocrine regulation of color and immunity, we find no evidence of associations between red coloration and ROS production. The granulocytes that are present in redder-abdomen males tend to be more phagocytic in males with redder abdomens, although this trend is again absent in Blackwater Lake (where spectrophotometry detects some, albeit reduced, red coloration).

The color-immune associations noted above involve multipe areas of the males’ bodies and different immune functions (e.g., red throat ~ granulocyte frequency; red abdomen ~ phagocytosis rates). This highlights an often-overlooked point that color-immune relationships entail highly multivariate association. Different body parts can be associated with different aspects of male immune function. Although there is evidence that stickleback females do use male color to evaluate immunity or parasite load [12], it is unclear to what extent they rely on different components of the multimodal color signals, and their relationship with multivariate immune variation. Future research in this area should examine the multi-channel nature of male color signals, rather than caricaturing stickleback color variation in terms of red throat color alone.

Our over-arching question here was whether male color-immune associations are consistent across populations that exhibit very different male colors and light environments. For some pairs of traits (abdomen brightness and phagocytosis rate), the color-immunity relationship appears to be fairly consistent across populations (e.g., no lake*color interactions) despite very different male colors. For other combinations of traits (preoperculum brightness and phagocytosis; red abdomens and phagocytosis, red surface area and proportion of granulocytes), we find support for lake*color interactions that would imply lake-specific color-immunity relationships. The most dramatic example of lake-specific color-immune correlations came from our photographic measures of color (S2 File). In predominantly red-throated Gosling and Lower Stella Lake populations, males with more extensive red on their throats had relatively fewer granulocytes (consistent with spectrophotometric results noted above). In contrast, Blackwater and Farewell fish lacked any red on their throats visible to our eyes; because there was no variance in red area in either melanic population, there was no correlation between red area and immune traits. More generally, our multivariate analyses (CCA) revealed significant color-immune relationships in the two red-throated male lakes, but not in the two melanic lakes (one assessed via photographic color measures only), despite equivalent statistical power.

Interestingly, although skin pigments convey no detectable information about immunity in Blackwater Lake, uniquely in this lake bluer eyes imply greater ROS production. This raises the intriguing question of whether, in melanic populations such as Blackwater, the loss of typical associations between immunity and red throat coloration leads to the evolution of female choice based on alternative characters, such as eye color, that may provide additional cues about male immune traits.

For evolution to alter color-immune relationships across lakes, both types of traits must be heritable. We have strong reason to believe this requirement is valid. First, several other studies have demonstrated that there is heritable variation in stickleback immune function [52, 58, 65], and color [37,38,66,67], despite known environmental effects on both kinds of traits [67–70]. In our own lab, we have used common-garden rearing experiments to confirm that there are heritable immunological differences between stickleback from Blackwater Lake and its adjoining stream (S7 Fig). In other common-garden studies, we found heritable differences (in the immune traits assayed here) between stickleback species pairs, between allopatric lakes, and between full-sib famlies from Gosling Lake (Weber, Bolnick, and Steinel, manuscript). Despite this heritable variation, it is also true that the traits measured here are highly labile. Stickleback color is known to change dynamically in response to the light environment, diet, social interactions, and stress. In particular, we see that red coloration declined with holding time (S3 File). Immune function also may change rapidly in response to stress, though we saw no effect of handling time. This leads to an essential caveat for our study: despite our best efforts to mitigate handling time effects, the color and immune traits we measured may differ, to an unknowable extent, from that of undisturbed individuals. However, our own qualitative observations confirm that wild nesting males exhibit extensive within-population variation in male color comparable to what we observed in captured males (e.g., Fig 1).

A second caveat is that the results presented here are entirely correlative, as it is not feasible at present to experimentally manipulate color (or immunity) in wild-caught stickleback. The goal here is to document, as much as possible, color-immune associations as they exist in the wild where they will be relevant to female mate choice decisions. It remains to be seen whether such associations persist in laboratory settings where they might be studied experimentally without the many confounding (but perhaps mechanistically important) variables such as diet, parasite loads, and social stresses. A corollary is that we cannot determine, with certainty, the direction of causation of associations presented here. We consider it likely that the link between color and immunity is mediated by variation in some unmeasured trait(s) such as hormone levels [49] that mechanistically links these phenotypes.

To conclude, we have shown that male color is correlated with various aspects of immune function in wild threspine stickleback. Some combinations of color-immune traits (e.g., granulocyte abundance versus red throat color) are correlated in a comparable manner in multiple populations. The repeatability of this trend, despite very divergent mean male colors, suggests that there may be some relatively immutable trade-off between these color and immune traits consistent with prior studies [49]. Such trade-offs are strong candidates for so-called ‘honest signals’ that females might use to evaluate male quality during mate choice.

Other color-immune combinations are inconsistently correlated across lakes. Melanic stickleback populations appear to have lost or mitigated the color-immune correlations that we observed in stereotypical red-male populations. Thus, consistent with our initial expectations, populations in different light environments, which are know to evolve different color patterns [39], also exhibit different color-immune relationships. Such flexible associations indicate that trade-offs between these traits are highly evolvable (e.g., evolution of the G matrix) or are environment-dependent, and therefore are not reliable cues of male quality. For example, increased availability of dietary carotenoids might allow individuals to both generate a bright red chin and mitigate oxidative damage from ROS production. We propose that the changes in color-immune associations, which we document here, might therefore alter patterns of sexual selection on male color, because of variable utility of color as a signal of male quality.

## Supporting Information

S1 Fig Side-welling irradiance at 1 meter depth differs between the four lakes examined here. (DOCX)

S2 Fig Variation in four aspects of immune function, within and among populations. (DOCX)

S3 Fig Variation in male color within and between lakes. (DOCX)

S4 Fig Summary of color-immune associations inferred from separate linear regressions, using spectrophotometric color measurements. (DOCX)

S5 Fig Summary of associations between parasite infections and either host immune traits or color, using spectrophotometric measures of color. (DOCX)

S6 Fig Summary of possible confounding variables’ effects on host immune traits or color, using spectrophotometric measures of color. (DOCX)

S7 Fig Heritable difference in immune function between two populations of stickleback reared in a common lab environment. (DOCX)

S1 File Supplementary methods describing immunological measurements. This includes 3 pages of text, and Figs A-C.
(DOCX)

S2 File Supplementary results of photographic analyses of male color variation and covariation with immune traits. This includes 6 pages of text, Figs A—N, and Table A.
(DOCX)

S3 File Supplementary results concerning potential confounding variables affecting color or immunity. This includes 9 pages of text, and Figs A—K, and Tables A-C.
(DOCX)

S1 Table Locations of the study populations. (DOCX)

S2 Table Results of linear models in which immune traits are tested for their association with total brightness of four male body parts, lake effects, and color by lake interaction effects. (DOCX)

S3 Table Results of linear models in which immune traits are tested for their association with color (proportion of orange-red reflectance) of four male body parts, lake effects, and color by lake interaction effects. (DOCX)
