# Supplementary material for: Population-Specific Covariation between Immune Function and Color of Nesting Male Threespine Stickleback
Source: PLoS One. 2015 Jun 3;10(6):e0126000. doi: 10.1371/journal.pone.0126000 (PMC4454680; doi:10.1371/journal.pone.0126000)
Supplement: S1 Fig — (DOCX) [file pone.0126000.s001.docx]

**Figure S1.** Side-welling irradiance at 1 meter depth differs between the four lakes examined here. Gosling and Lower Stella Lakes transmit more light overall (higher sidewelling irradiance) than Blackwater or Farewell Lakes. Normalizing for total irradiance, to better visualize wavelength composition, reveals that side-welling light in both Gosling and Lower Stella Lakes contains a higher proportion of low-wavelength (bluer) light and less long-wavelength (redder) light. Thus, males with redder throats will tend to look brighter in Gosling and Lower Stella (more light to reflect) and will contrast more with the side-welling light (which is less red). Irradiance values were measured with a StellarNet spectrometer, at 1 meter depth, with the
