# Supplementary material for: Population-Specific Covariation between Immune Function and Color of Nesting Male Threespine Stickleback
Source: PLoS One. 2015 Jun 3;10(6):e0126000. doi: 10.1371/journal.pone.0126000 (PMC4454680; doi:10.1371/journal.pone.0126000)
Supplement: S2 Fig — (DOCX) [file pone.0126000.s002.docx]

**Figure S2.** Variation in four aspects of immune function, within and among populations. Population means are indicated with larger points with one standard error bars. The percentage of granulocytes is higher in Farewell and Lower Stella stickleback than in Gosling, which in turn has more granulocytes than Blackwater stickleback (panel A, ANOVA F_3,135_ = 4.0, P = 0.009). The activity level of these cells also differs between populations (panel B). Median ROS burst is highest in Blackwater, and 35%, 75% and 95% lower in Gosling, Lower Stella, and Farewell respectively (F_3,135_ = 12.5, P < 0.001). This among-lake variation remains significant (F_3,132_ = 4.5, P < 0.001) after accounting for confounding effects of cell abundance (F_1,132_ = 21.6, P < 0.001). On average, 69%, 52%, 39%, and 38% of granulocytes were phagocytic, in fish from Lower Stella, Farewell, Gosling, and Blackwater Lakes, respectively (panel B, F_3,138_ = 24.9, P < 0.001), and this remains significant after accounting for cell abundance (ANOVA cell count F_1,125_ = 48.04, P < 0.001, lake F_2,125_ = 18.2, P < 0.001).
