# Supplementary material for: Population-Specific Covariation between Immune Function and Color of Nesting Male Threespine Stickleback
Source: PLoS One. 2015 Jun 3;10(6):e0126000. doi: 10.1371/journal.pone.0126000 (PMC4454680; doi:10.1371/journal.pone.0126000)
Supplement: S3 Fig — (DOCX) [file pone.0126000.s003.docx]

**Figure S3. Variation in male color within and between lakes.** Each point represents the proportion of red, blue, and green reflectance (normalized by total reflectance) for a given male. Symbols and colors as in Fig. 2 of the main text. Each ternary plot represents the color variation for a different part of the body. A= lower eye (Pillai = 0.826, df = 2, P < 0.001 ), B = preoperculum (Pillai = 0.502, df = 2, P < 0.001 ), C = throat (Pillai = 0.658, df = 2, P < 0.001), D = abdomen (Pillai =0.346, df = 2, P < 0.001 ). The triangle at the upper left shows the extent to which the main ternary plots are zoomed into the larger color space.
