# Supplementary material for: Population-Specific Covariation between Immune Function and Color of Nesting Male Threespine Stickleback
Source: PLoS One. 2015 Jun 3;10(6):e0126000. doi: 10.1371/journal.pone.0126000 (PMC4454680; doi:10.1371/journal.pone.0126000)
Supplement: S4 Fig — (DOCX) [file pone.0126000.s004.docx]

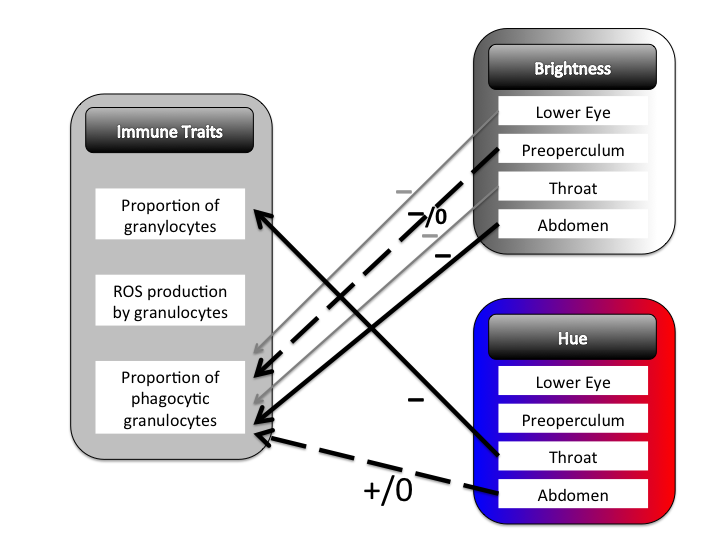


**Figure S4.** Summary of color-immune associations inferred from separate linear regressions, using spectrophotometric color measurements. Solid dark lines represent significant main effects of color on a given immune trait. Dashed lines represent significant lake by color interactions, indicating population-specific color-immune relationships. Pale lines represent marginally significant main effects. Plus or minus signs indicate effect direction. For lake*color interactions, we indicate whether the interaction occurs because of opposite effect directions (-/+) or because effects are present in some and absent in other lakes (e.g., -/0). For purposes of effect directions, redder males have higher hue scores.
