# Supplementary material for: Population-Specific Covariation between Immune Function and Color of Nesting Male Threespine Stickleback
Source: PLoS One. 2015 Jun 3;10(6):e0126000. doi: 10.1371/journal.pone.0126000 (PMC4454680; doi:10.1371/journal.pone.0126000)
Supplement: S5 Fig — (DOCX) [file pone.0126000.s005.docx]

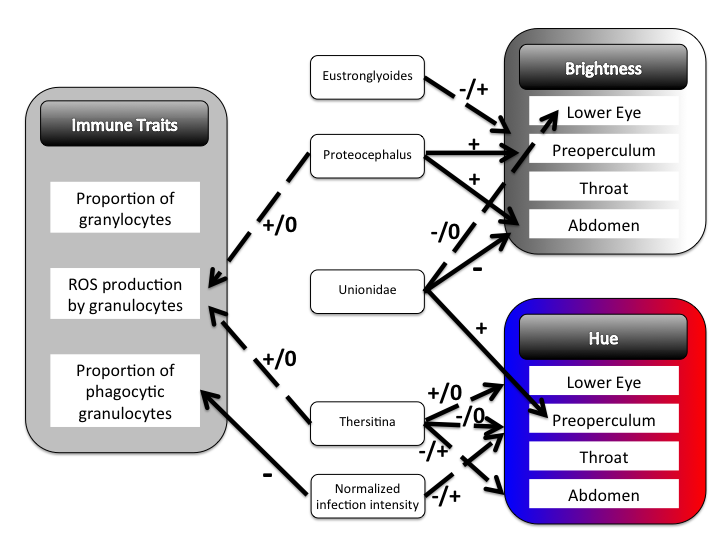


**Figure S5.** Summary of associations between parasite infections and either host immune traits or color, using spectrophotometric measures of color. See Supplementary Text 3 for details. As in Fig. S4, solid lines represent significant main effects and dashed lines represent lake-specific effects (e.g., significant lake by infection interactions), from separate linear models for each comparison of infection state and color or immunity. Infection variables (boxes in the center column of the figure) were treated as independent variables in the linear regression, although particularly for immune function the cause-effect relationship could arguably be reversed. For simplicity, parasites without any significant effects are omitted from this figure. Plus or minus signs indicate effect direction. For lake*color interactions, we indicate whether the interaction occurs because of opposite effect directions (-/+) or because effects are present in some and absent in other lakes (e.g., -/0). For purposes of effect directions, redder males have higher hue scores.
