# Supplementary material for: Population-Specific Covariation between Immune Function and Color of Nesting Male Threespine Stickleback
Source: PLoS One. 2015 Jun 3;10(6):e0126000. doi: 10.1371/journal.pone.0126000 (PMC4454680; doi:10.1371/journal.pone.0126000)
Supplement: S6 Fig — (DOCX) [file pone.0126000.s006.docx]

**
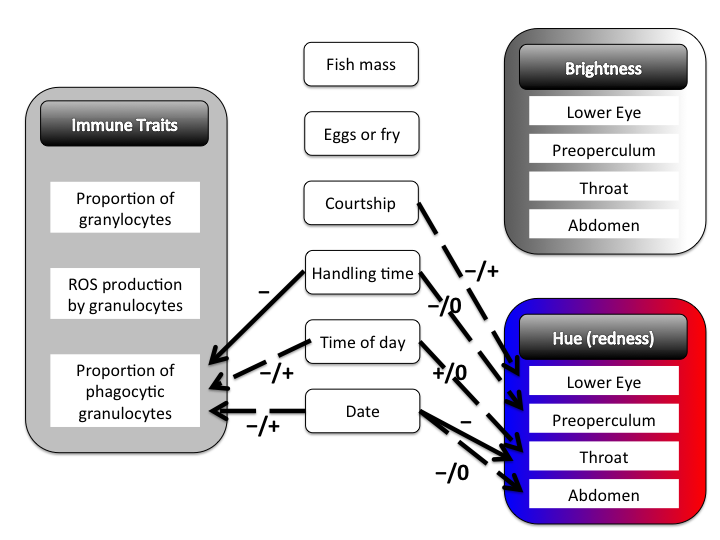
**

Figure S6. Summary of possible confounding variables’ effects on host immune traits or color, using spectrophotometric measures of color. See Supplementary Text S3 for details. As in Fig. S4, solid lines represent significant main effects and dashed lines represent lake-specific effects (e.g., significant lake by variable interactions), from separate linear models for each comparison. Plus or minus signs indicate effect direction. For lake*color interactions, we indicate whether the interaction occurs because of opposite effect directions (-/+) or because effects are present in some and absent in other lakes (e.g., -/0). For purposes of effect directions, redder males have higher hue scores.
