# Supplementary material for: Population-Specific Covariation between Immune Function and Color of Nesting Male Threespine Stickleback
Source: PLoS One. 2015 Jun 3;10(6):e0126000. doi: 10.1371/journal.pone.0126000 (PMC4454680; doi:10.1371/journal.pone.0126000)
Supplement: S7 Fig — (DOCX) [file pone.0126000.s007.docx]

**Figure S7. Heritable difference in immune function between two populations of stickleback reared in a common lab environment.** In a pilot study testing our immune measurement protocols, we assayed immune traits in laboratory-reared stickleback derived from two populations: Blackwater Lake and its inlet stream (100 m upstream from the lake). Fish from both habitats were reared from eggs in a parasite-free laboratory aquarium environment, with identical water conditions and diet. Despite this common-garden rearing, lake and stream stickleback differed significantly for some aspects of immune function. Twenty families of stickleback from Blackwater Lake, and twenty from the inlet stream to Blackwater were generated by in vitro fertilization between wild-caught parents. In the summer of 2009, eggs were transported to the University of Texas, hatched, and reared to adulthood, fed freshly hatched brine shrimp and later pellet food and freeze dried bloodworms. In late spring 2010, a sample of individuals drawn from the families were measured for immune function as described in SI Text S1, measuring relative abundance of granulocytes and lymphocytes, and ROS burst. A binomial general linear model confirmed that the ratio of granulocytes to lymphocytes differed between populations (t = 2.93, P = 0.0058, df = 36), as plotted in this figure. Data points are individual fish (one per family), and sample mean and standard error bars are plotted to the left of the scatter of data points. The populations did not differ in mean total cell count (P = 0.705), or ROS production (F_1,35_ = 0.887, P = 0.353). While this is not evidence for trait heritability within or between our four focal populations in this study, it does confirm that some of the immune traits can be partly heritable within one of the study sites (Blackwater lake and its adjoining stream), and thus likely to exhibit heritable variation within and between populations.
