# Supplementary material for: Population-Specific Covariation between Immune Function and Color of Nesting Male Threespine Stickleback
Source: PLoS One. 2015 Jun 3;10(6):e0126000. doi: 10.1371/journal.pone.0126000 (PMC4454680; doi:10.1371/journal.pone.0126000)
Supplement: S1 File — This includes 3 pages of text, and Figs A-C. (DOCX) [file pone.0126000.s008.docx]

**S1 File**

**Supplementary Methods Describing Immunological Measurements**

Cell suspensions of head kidneys (HK) were obtained by gently macerating HKs with a pipet tip, then straining through a 35 um BD Falcon nylon screen into 2 mL cold R-90 culture medium (90% (v/v) RPMI 1640 (+L-Glutamine, -Phenol red; Gibco) + 10% (v/v) distilled water). The suspension was washed three times in 2ml cold R-90 (10 minutes at 550 g at 4° C) and resuspended in 0.5 mL. We carried out three assays on the resulting cell suspension, measuring (i) the relative abundance of granulocytes and lymphocytes as shown in Figure A in S1 File, (ii) ROS production as shown in Figure B in S1 File, and (iii) phagocytosis rates as shown in Figure C in S1 File.

To measure cell composition and ROS production, we created three assays per fish (each assay done in triplicate): (i) as a control and to count cells, we assayed the cell suspension alone, (ii) to measure basal ROS production we treated cells with 10 μg/ml dihydrorhodamine 123 (DHR, Sigma) in DMSO, and (iii) to measure ROS production we treated cells with both DHR and 15 μl of 1 μg/ml Phorbol 12-myristate 13-acetate (PMA, Sigma) to stimulate cells, with final solution concentrations of 0.13 ng/μl PMA and 2 ng/μl DHR. DHR acts as an indicator for ROS production, by diffusing across membranes into cells where it is oxidized to cationic rhodamine 123, which fluoresces at 536nm. All three assays received 20 μl of cells. A control assay involved cell culture alone, while assays (ii) and (iii) received 20 μl of DHR and all samples received enough R-90 to bring total volume up to 100 μl. All cell cultures were incubated at 18° C and 2% CO_2_ for 10 minutes, then either 15 μl of PMA (assay iii) or 15 μl of R-90 (assays i and ii) were added. Assays were incubated for an additional 20 min, then placed on ice with 100 μl ice cold R-90 to stop the reaction, and 0.2 μl propidium iodide (PI; 1 mg/ml in water, Sigma; final concentration 0.93 ng/μl) added to stain dead cells.

The cells were then analyzed on an Accuri C6 flow cytometer and 50,000 events were collected per sample. The number of live cells within that 50,000-event sample depends on head kidney size, cell viability, and the number of additional particles of debris, but typically ranged from ~500 to ~ 20,000 cells. The Accuri measures cell size, dimensions via light scatter through the sample as well as fluorescence on several color channels. We used forward- and side-scatter to classify cells as granulocytes (which tend to be large and have complex internal membranes and thus have high forward- and side-scatter) or lymphocytes (which tend to be small and little internal membrane complexity and so have low forward- and side-scatter). Cellular debris was discarded based on size, and dead cells (PI stained) were gated out as well. Lymphocyte and granulocyte counts were used to quantify the frequencies of granulocytes in the tissue (Figure A in S1 File).

Next, we used the intensity of DHR fluorescence as a measure of ROS activity within each cell, and calculated median intensity of fluorescence across all viable cells in a sample. We quantified two metrics of ROS production. Basal ROS levels were measured as the difference in median fluorescence between unstimulated (assay ii) and control (assay i) cells. ROS production in response to mitogen stimulation by PMA was measured as the difference in median fluorescence between stimulated (assay iii) and unstimulated (assay ii) cells (Figure B in S1 File). We repeated these measures gating only on granulocytes in order to obtain a granulocyte-specific measure of ROS production, to ensure our results were not dependent on granulocyte:lymphocyte ratio. We focus our analyses on ROS production by PMA-activated granulocytes. All flow cytometry measures were calculated using the flowCore Bioconductor package in R, and custom R scripts available upon request.

To measure phagocytosis rates, we set up three assays per fish (each in triplicate): (i) cells alone, (ii) cells with red polychromatic beads (Polysciences) incubated at 4° C, and (iii) cells with beads incubated at 18° C and 2% CO_2,_ where cells are more active. Cells that have internalized beads subsequently fluoresce red (Figure C in S1 File). Each assay received 20 μl of cells, plus either 80 μl R-90 (assay i) or 20 μl beads at 10^7^ beads/ml plus 60 μl R-90 (assays ii and iii). All assays were incubated for 19.5 hours at 4° C or 18° C (assay i & iii), at which time they were transferred to ice and 100 μl ice-cold R-90 added to stop reactions, and 0.2 μl of 1 mg/ml PI added. The samples were immediately measured on the Accuri C6 flow cytometer. After gating out solitary beads, small debris (very low FSC) and dead (PI+) cells, we used red fluorescence associated with cells as a marker of phagocytosis. Red fluorescence occurred in discrete peaks that likely correspond to integer numbers of beads phagocytized, but because we do not have direct confirmation that these discrete peaks represent numbers of beads, we focus on measuring the proportion of granulocytes that acquired at least one bead. We do see phagocytosis by lymphocytes as well, but to avoid confounding granulocyte:lymphocyte ratio with phagocytosis measures, we focus just on the subset of cells inferred to be granulocytes based on forward- and side-scatter.

**
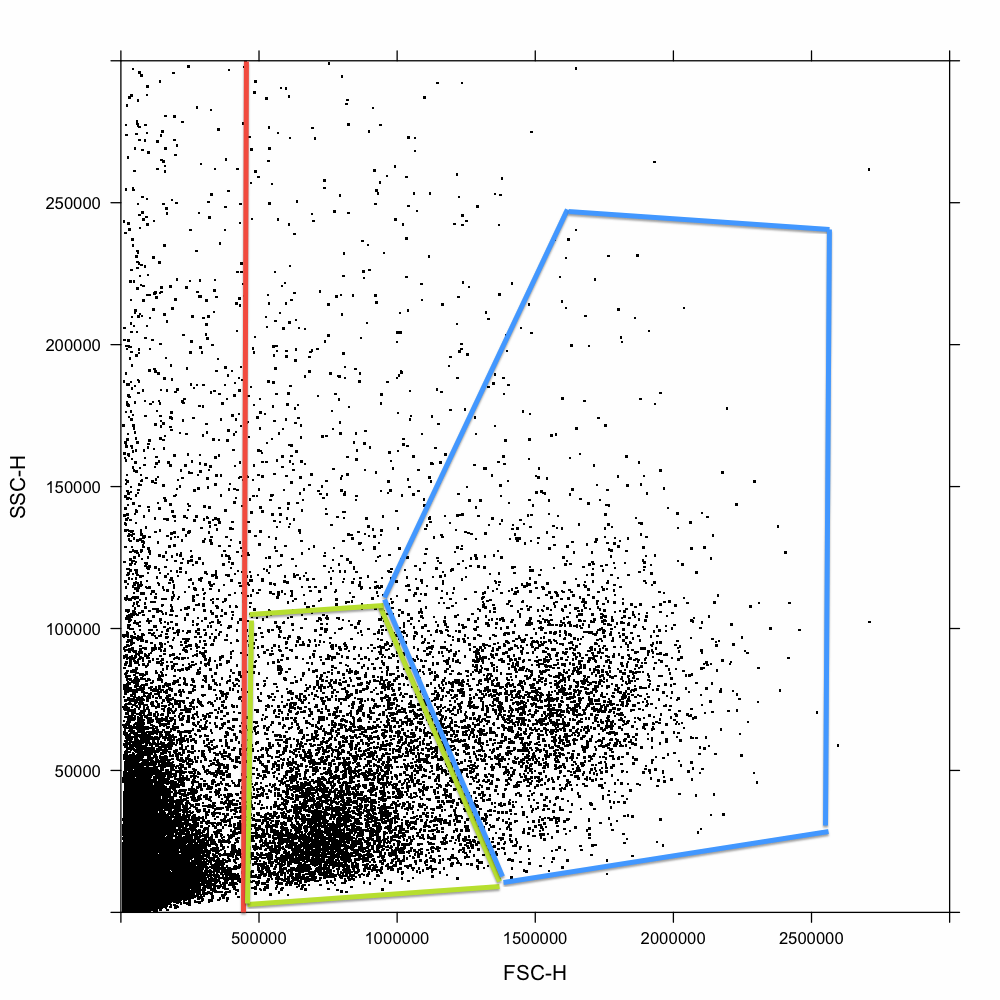
**

**Figure A in S1 File. Example of flow cytometry estimation of the relative abundance of lymphocytes and granulocytes**. Here data are presented from a single arbitrarily selected fish. Here we provide a detailed explanation of this plot for readers not familiar with flow cytometry. The Accuri C6 flow cytometer was set to sample 50,000 particles and measure forward scatter (FSC-H) and side scatter (SSC-H) of each cell or particle sampled. Forward scatter is positively correlated with cell size, side scatter is positively correlated with internal membrane complexity. Very small particles which fall to the left of the vertical red line (FSC-H <45,000) are inferred to be cellular debris and sub-cellular particles. Events to the right of the vertical red line are considered to be cells. Lymphocytes such as B- and T-cells are typically small with little subcellular structure, and so have low FSC and SSC (green polygon gate). Granulocytes are typically (but not always) larger and exhibit more complex membrane structures, and so have higher FSC and SSC (blue polygon gate). There are two distinct peaks of cell abundance, one for lymphocytes and one for granulocytes. Rather than define arbitrary gates, we used the *mclust* package in R to implement a Bayesian clustering analysis delineating two clusters of cells (excluding debris and PI^+^ dead cells). In all cases two clusters fit the data significantly better than one cluster. We then obtained each cell’s posterior probability of membership in each cluster, and summed these posterior probabilities across all cells to obtain estimates (weighted by assignment uncertainty) of the proportion of lymphocytes or granulocytes within the sample. These measures were done in triplicate on independent samples from the cell suspension, and the three estimates averaged. Categorization of cell types by forward and side-scatter has been validated by visually inspecting microscopic images of cells from each gate, using an ImageStream flow cytometer (Natalie Steinel, manuscript in preparation). The ImageStream measures forward and side-scatter on each cell, as well as photographing each cell in multiple color channels that allow visualization of cell size, shape, granularity, and nucleus morphology.

**Figure B in S1 File. Example of how we calculated ROS production from a given stickleback**. The example presented here is from a single arbitrarily selected individual. Cell suspension aliquots were sampled for green fluorescence (intensity per cell) either using untreated control cells (grey histogram), cell suspension with DHR added (blue histogram) to fluoresce after reacting with ROS, or DHR plus PMA (an immunostimulant to induce ROS burst, red histogram). The difference between median fluorescence intensity for DHR versus control cells represents the background quantity of ROS (blue versus grey arrows). The difference between median fluorescence for DHR^+^ PMA^+^ versus DHR^+^ (red versus blue arrows) measures ROS burst, and is our focal metric here. These measures may be done on all live cells in aggregate, or after gating to focus on granulocytes or lymphocytes alone. In this manuscript we focus our analyses exclusively on granulocyte ROS production following PMA stimulation.

**
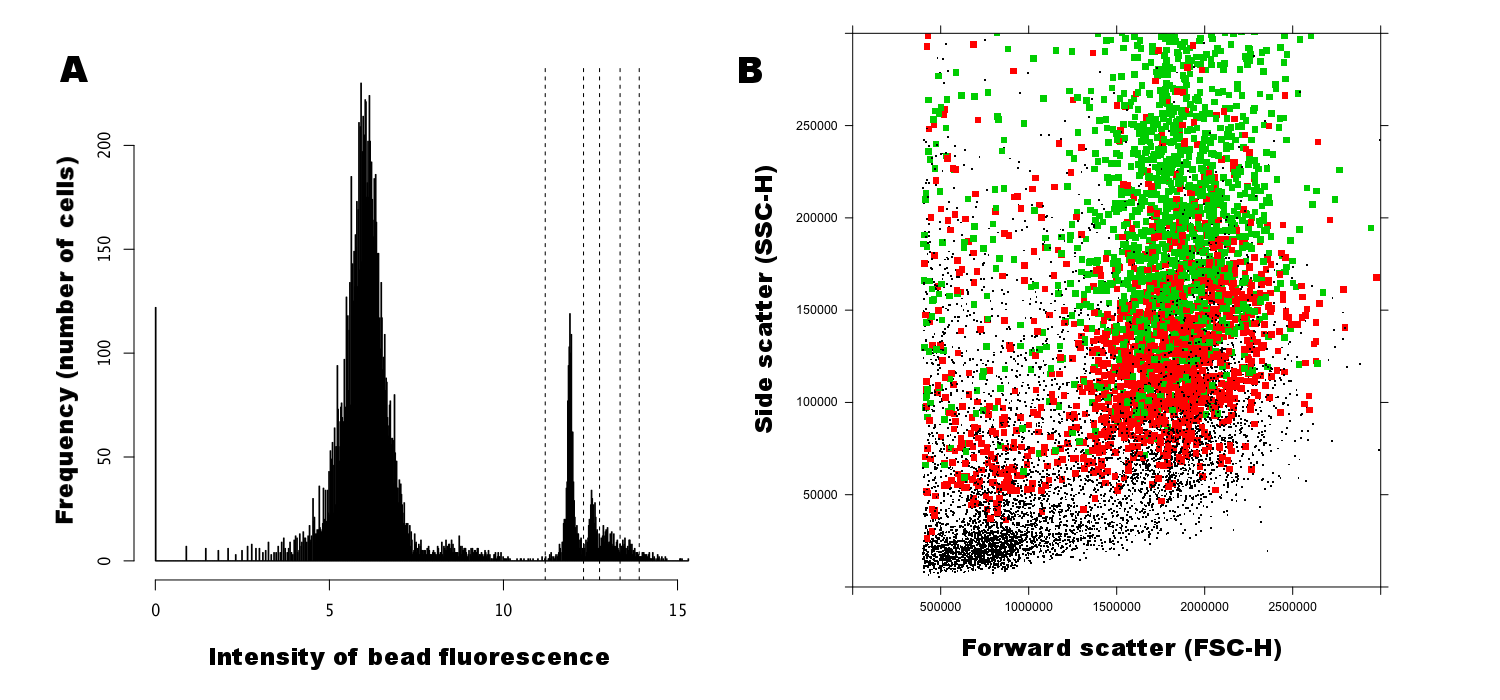
**

**Figure C in S1 File. An illustration of calculating phagocytosis rates from a single stickleback**. Unphagocytized beads are small and removed by the debris gate, but can be distinguished from other debris by their intense red fluorescence. Unphagocytized beads and cellular debris were excluded based on size. Cells which had internalized beads were detected by their red fluorescence. Focusing on cells, we (A) generated a histogram of bead fluorescence intensity. The large peak representing the majority of cells is present in both bead-negative controls and bead treatments, and represents the background fluorescence of the cells. Dead cells (PI^+^) typically have fluorescence intensities from 8 to 10 (very low broad secondary peak immediately to the right of the majority of cells, and could be found in both bead-negative and bead-treatment assays. Cells that phagocytized beads appear as one or more sharp peaks of cell counts between 11 and 15 fluorescence units (two major peaks in panel A), these peaks separated by vertical dashed lines in the figure. The divisions between peaks were automatically imputed using a normal distribution mixture model. We infer, but cannot conclusively prove at this point, that the distinct peaks of bead fluorescence represent discrete numbers of phagocytized beads (1 bead, 2 beads, etc.). B. Having identified which cells have phagocytized one or more beads, we next used forward and side-scatter (FSC-H and SSC-H) to identify cells as lymphocytes or granulocytes (as illustrated in Figure A in S1 File). Here we plot cells’ FSC and SSC values, and mark bead^+^ cells with larger square symbols. Red squares are cells inferred to have taken up one bead, green are inferred to have phagocytized two or more beads. Note that the majority of granulocytes are phagocytic, but the majority of lymphocytes are not. To avoid confounding our phagocytosis assay with variation in relative abundances of the cell types, we calculated the fraction of phagocytic cells (bead^+^) within the granulocyte gate, excluding lymphocytes. Repeating analyses in the main paper using all cells’ phagocytosis rates, or the mean number of beads phagocytized, yields qualitatively equivalent results, so our results are robust to our choice of ways to quantify phagocytosis. Note also that only recently have immunologists begun to appreciate that B cells and other lymphocytes have phagocytic activity, albeit at lower rates than granulocytes [61].
