# Supplementary material for: Population-Specific Covariation between Immune Function and Color of Nesting Male Threespine Stickleback
Source: PLoS One. 2015 Jun 3;10(6):e0126000. doi: 10.1371/journal.pone.0126000 (PMC4454680; doi:10.1371/journal.pone.0126000)
Supplement: S2 File — This includes 6 pages of text, Figs A—N, and Table A. (DOCX) [file pone.0126000.s009.docx]

**S2 File**

**Supplementary Results of Photographic analyses of Male Color Variation**

**and Covariation with Immune Traits**

To compensate for the loss of spectrophotometric color data from one lake, we also measured color from photographs of all fish. Within one minute after spectrophotometer measurement, each male was photographed inside an opaque container, using a Cannon Rebel SLR camera with a diffused flash (Sigma EF-530 DG Super) with programmable fixed light output and a fixed exposure time and aperture. The flash light source provided constant illumination and spectral composition. Importantly, light output does not vary even as the battery is discharged (the flash will not fire if battery power is insufficient). Each photograph included a color standard (X-Rite ColorChecker Card). Brightness and hue of these standards did not vary appreciably across photographs, confirming the constant illumination quality provided by the fixed-output flash. That is, we evaluated total brightness (R + G + B brightness) in GIMP ([www.gimp.org](http://www.gimp.org)) for the white, middle-grey, and black squares on the card of each photograph. We also evaluated redness (R / (R+G+B)) of the reddest square and blueness (B/(R+G+B)) of the bluest square. Each photograph’s color standard was measured in triplicate. We did not find significant among-photograph variation in color or brightness, consistent with using a single constant-illumination flash.

*Measuring color.*

Images including the fish specimen and a color standard were imported into GIMP (www.gimp.org). We then defined a threshold intensity of orange-red, and used GIMP to outline and measure the area of the throat that exceeded that threshold (top panel of Figure A in S2 File). The area of red was divided by the area of the specimen to obtain a relative red area score. We next drew a rectangle whose right and left edges corresponded to the right and left edge of the eye, and whose top and bottom edges corresponded to the bottom of the eye and the lower jaw respectively (lower panel of Figure A in S2 File). We placed a similar-sized rectangle on the preoperculum, located such that the top left corner of the rectangle was on the top of the upper origin of the operculum. We placed a similar-sized rectangle on the abdomen, such that the lower right corner was on the origin of the pectoral spine. The mean intensities of red, blue, and green (RGB) within each rectangle were recorded. Brightness was measured by the sum of these RGB values. Redness was measured by dividing the red-channel intensity by the summed intensity across all three channels (R / RGB).

*Color variation among lakes*

As with spectrophotometric measures of color, we observed significant variation in color between populations. First considering the relative area of the red throat, we found that the red throat of Lower Stella fish was nearly twice as large (adjusting for body size) as in Gosling fish (Figure B in S2 File; t = 4.13, P = 0.0001). In both Blackwater and Farewell lakes, not a single sampled male had detectable red throats. Considering RGB values of the three body parts assayed, a MANOVA again confirmed significant color variation between populations (Pillai’s trace = 1.4 , P < 0.0001; Figure C in S2 File). Analyzing each body part separately, populations were significantly different (P < 0.0001) for every body part, although the population effect size was greatest for the preoperculum (F_9,414_ = 16.15; variation ranges from very red in Lower Stella to black in Blackwater) and least for the abdomen (F_9,414_ = 10.15), with an intermediate variance explained for the throat (F_9,414_ = 13.74).

*Relationship between photographic and spectrophotometric color measurements*

The spectrophotometric measures of color provide a very detailed description of the full spectrum of color reflectance, including UV, at very specific small locations on the body. We did the spectrophotometer measurements in triplicate at slightly different locations within a body region, to average over small-scale color variation. In contrast RGB values provide a very coarse measure of color averaged over a much larger area, while red area provides a measure of the spatial extent of a signal. These therefore give complementary and non-identical measures of color. Accordingly, there was a complex and sometimes population-specific relationship between photograph and spectrophotometric color. For example, the area of the red throat in photographs is only modestly correlated with the intensity of red reflectance at small points on the throat (Figure D in S2 File, r = 0.625 overall). The proportion of R (in RGB values) of the each body part is correlated with the reflectance of red in the corresponding body part, but this relationship is driven mostly by between-population differences. In linear models with lake, color, and lake*color interactions, for two of the three body parts we detect significant interactions indicating population-specific relationships between photographic and photometric measures of color. (Figures E & F in S2 File) and brightness (Figure G in S2 File).

*Color-immunity associations from photographic color measurements*

As a first pass analysis we used canonical correlation analyses (CCA) to test for multivariate associations between all three immune parameters, and the seven photographic color measures (red area, and brightness and hue of each of three body parts). We observed significant canonical correlations in Gosling Lake (P = 0.0002) and Lower Stella Lake (P < 0.0001), the two lakes with predominantly red throated males (Table A in S2 File). There was a marginally significant canonical correlation in Blackwater Lake (melanic males; P = 0.0743), and no significant association in Farewell. The two red-male lakes’ CCA results are robust to multiple comparison corrections. We therefore infer that color-immune associations are restricted to red-male lakes, consistent with our findings from spectrophotometric data. Because of this inconsistent effect (and different factor loadings) across lakes, there was no overall significant CCA using all four lakes together (P = 0.411; Table A in S2 File). As with the spectrophotometric CCA results, hue rather than brightness explained more immunological variation. Granulocyte frequency and phagocytosis contributed more strongly to the canonical correlations than did ROS production.

As with the spectrophotometric measures of color, we also observe associations between immune traits and photographic color measures when using linear regressions of pairs of traits. Typically these associations are population-specific. For example, we observe a negative correlation between the proportion of granulocytes and the extent of red on males’ throats (Figure H in S2 File). This trend is highly parallel in Gosling and Lower Stella Lakes, with the result that there is a significant effect of red area (P = 0.041) on granulocyte abundance, but no significant effect of lake (P = 0.111) or lake*red interaction (P = 0.495). However, Farewell and Blackwater fish completely lack red throats, so this trend is necessarily absent within those lakes. The same trend holds for red intensity of the throat (divided by summed RGB values) in the two red-male lakes (Figure I in S2 File; red intensity P = 0.0049, Lake P = 0.0164; considering each lake separately, Lower Stella r = -0.4719, P = 0.0064; Gosling r = -0.1731, P = 0.2918). In contrast Blackwater and Farewell fish exhibit no significant trends. Phagocytosis rates by granulocytes also increased with red intensity of the preoperculum (in Gosling Lake only, Figure J in S2 File) and abdomen (not significant in any single lake, but the trend holds across all lakes, Figure K in S2 File).

Examining principal components of RGB scores of all three body parts together, again reveals lake-specific color-immunity associations. In Lower Stella Lake alone, color PC2 is significantly associated with granulocyte abundance (Figure L in S2 File). The same color PC axis was significantly associated with changes in granulocyte phagocytosis rates in both Lower Stella and Gosling lakes, albeit in opposing directions resulting in a significant color by lake interaction (Figure M in S2 File).

*Conclusion*

In both Lower Stella and Gosling Lakes, male stickleback exhibit a distinctive red-throat that is considered typical of stickleback. In these two lakes, multiple color traits were significantly associated with multiple immune traits (mostly granulocyte relative abundance and phagocytosis rate, rarely ROS production; all significant effects are summarized in a diagram Figure N in S2 File).

The lack of ROS effect on photographic color is consistent with the absence of such effects with spectrophotometric data. The associations between photographic color and immune traits are sometimes but not always comparable between the two populations. For some color-immune associations the trend is restricted to just one population (usually Lower Stella), or even in opposite directions. Thus, even in red-throated populations the relationship between color and immunity is heterogeneous. In comparison, the two populations without noticeable red throats (Blackwater and Farewell) exhibited no significant color-immune relationships when measuring color from photographs or by spectrophotometer. We therefore believe that the core conclusion of our paper is robust to our method(s) of quantifying color. Namely, in predominantly red populations male color is correlated with immune function and therefore could act as a signal of male quality. In contrast, in melanic populations this relationship is lost and color ceases to be an effective indicator of immunity (at least for the traits measured here).

**Figure A in S2 File. Illustration of the two approaches to measuring color from photographs**: (top) area of red/orange as a proportion of body area, and (bottom) RGB values were scored within rectangles placed on the abdomen, preoperculum, and throat. The perimeter of the red area (top) was scored by eye, by two independent observers.

**Figure B in S2 File. Histogram of the extent of red area (relative to body area) in fish from four lakes**. Histogram bars are overlapped rather than vertically stacked. Bblue bars for Lower Stella Lake, green bars for Gosling Lake, and grey bar for Blackwater Lake, black for Farewell Lake. The means of Lower Stella and Gosling are indicated by blue and green arrows respectively. For Blackwater and Farewell lake fish, all individuals have values of zero.

**Figure C in S2 File. Variation in male color within and among populations, as measured from photograph GRB scores.** Distribution of specimens along the first and second principal component axes of color values (RGB) obtained from three body parts (throat, peroperculum, abdomen). Symbols and colors distinguish lakes, as in S2 Figure (red: Farewell Lake, blue: Lower Stella Lake, green: Gosling Lake, black: Blackwater Lake).

**Figure D in S2 File. Comparing photographic versus photometric throat color.** The relationship between the relative area of the red throat in photographs, and the reflectance of red-orange wavelengths at small points on the throat. The relationship is unavailable for Farewell Lake, where spectrophotometric data was corrupted. The relationship is undefined in Gosling Lake where there is no variation in red area (all zero). The relationship is significant in Gosling (P = 0.00006) but not Lower Stella (P = 0.458), although in a linear model with both lakes there is no significant lake by red area interaction (P = 0.14) indicating that the overall effect of color (P = 0.004) may not differ between lakes. Symbols and colors as in S2 Figure (blue: Lower Stella Lake, green: Gosling Lake, black: Blackwater Lake). We include this here because comparisons of photographic versus spectrophotometric measures of color are rarely published, but are methodologically important to be aware of.

**Figure E in S2 File. Comparing photographic versus photometric throat color**. Association between spectrophotometric and photographic measures of throat redness. The correlation between these ostensibly redundant measures is significant in Blackwater lake (P = 0.0065) and Gosling Lake (P < 0.0001) but not Lower Stella (P = 0.8769), with the result that there is both an overall association between the measures of brightness (P < 0.0001), a lake effect (P < 0.0001), and an interaction (P = 0.041). There is almost an identical pattern for preoperculum redness, so we do not reproduce the redundant figure here. Symbols and colors as in S2 Figure (blue: Lower Stella Lake, green: Gosling Lake, black: Blackwater Lake).

**Figure F in S2 File. Comparing photographic versus photometric abdomen color.** Spectrophotometric and photographic measures of abdomen redness agree closely (overall r = 0.598, P < 0.001). Taking lake into account as well, we find significant positive associations within Blackwater (P = 0.0095) and Gosling (P < 0.0001) but not Lower Stella (P = 0.1365), although the trend is similar in all three lakes (hence an overall main effect of color P < 0.0001 but no significant lake by color interaction, P = 0.826). Symbols and colors as in S2 Figure (blue: Lower Stella Lake, green: Gosling Lake, black: Blackwater Lake).

.

**Figure G in S2 File. Comparing photographic versus photometric brightness.** Spectrophotometric and photographic measures of male brightness agree for both abdomen and preoperculum, but not throat (not shown, r = 0.17, P = 0.0775). For the abdomen, males with higher RGB totals also had larger areas under the reflectance curve (color P = 0.018), though there was also an effect of lake (P = 0.0067) and no interaction (P = 0.134). For the preoperculum, there was a strong overall association between RGB and curve area (P < 0.0001) as well as a lake effect (P < 0.0001) and a lake by RGB interaction (P = 0.0163) because the positive association was significant in Gosling and Blackwater but not Lower Stella. Symbols and colors as in S2 Figure (blue: Lower Stella Lake, green: Gosling Lake, black: Blackwater Lake).

**Figure H in S2 File. Chin red area is negatively correlated with granulocyte relative abundance in two lakes**. The association is not significant in either lake alone (0.1 > P > 0.05), but the trend is significant when pooling the lakes (P = 0.04, including a lake effect). There is no interaction effect between lake and color, because the slopes from Lower Stella and Gosling are so parallel. Blackwater and Farewell fish are not used in the model because there is no variance in the x axis variable (all males had no red on chin). Symbols and colors as in S2 Figure (red: Farewell Lake, blue: Lower Stella Lake, green: Gosling Lake, black: Blackwater Lake).

**Figure I in S2 File. The relative intensity of red on males’ throat is negatively correlated with granulocyte frequency in one lake.** Association between the relative intensity of red on the throat, and the relative abundance of granulocytes. The association is significant in Lower Stella Lake (solid line, P = 0.0064), but in no other lake. There is a main effect of both color (P = 0.0291) and lake (P = 0.0070) but no interaction (P = 0.2588). Symbols and colors as in S2 Figure (red: Farewell Lake, blue: Lower Stella Lake, green: Gosling Lake, black: Blackwater Lake).

**Figure J in S2 File. Redder preoperculum is associated with phagocytosis rates in one lake and overall.** Association between preoperculum red intensity and granulocyte phagocytosis rates. The association is significant only in Gosling Lake (P = 0.0289), but there is a main effect of preoperculum redness (P = 0.0390) and lake (P = 0.0047), though no significant interaction (P = 0.1148). Symbols and colors as in S2 Figure (red: Farewell Lake, blue: Lower Stella Lake, green: Gosling Lake, black: Blackwater Lake).

**Figure K in S2 File. Redder-abdomen males have higher phagocytosis rates.** Association between abdominal red intensity and granulocyte phagocytosis rates. The association is not significant in any one lake alone (dashed lines), but there is a main effect of abdominal redness (P = 0.0074) indicated by the grey trend line, as well as a main effect of lake (P < 0.0001) and no interaction between redness and lake (P = 0.2288). Symbols and colors as in S2 Figure (red: Farewell Lake, blue: Lower Stella Lake, green: Gosling Lake, black: Blackwater Lake).

**Figure L in S2 File. Association between the second principal component axis of RGB color, and the relative abundance of granulocytes**. The association is significant in Lower Stella alone (r = 0.5476, P = 0.0019, solid line), and essentially flat in the other three lakes resulting in no main effect of color PC2 (P = 0.0753), but a main effect of lake (P = 0.0127) and a lake*PC2 interaction (P = 0.0433). Symbols and colors as in S2 Figure (red: Farewell Lake, blue: Lower Stella Lake, green: Gosling Lake, black: Blackwater Lake).

**Figure M in S2 File. Association between the second principal component axis of RGB color, and phagocytosis rates by granulocytes.** The association is marginally significant in Lower Stella alone (P = 0.0408, solid blue line), and significant in the opposite direction in Gosling (P = 0.0409), resulting in a significant lake by PC2 interaction (P = 0.0373) but no main effect of PC2 (P = 0.4340). Symbols and colors as in S2 Figure (red: Farewell Lake, blue: Lower Stella Lake, green: Gosling Lake, black: Blackwater Lake).


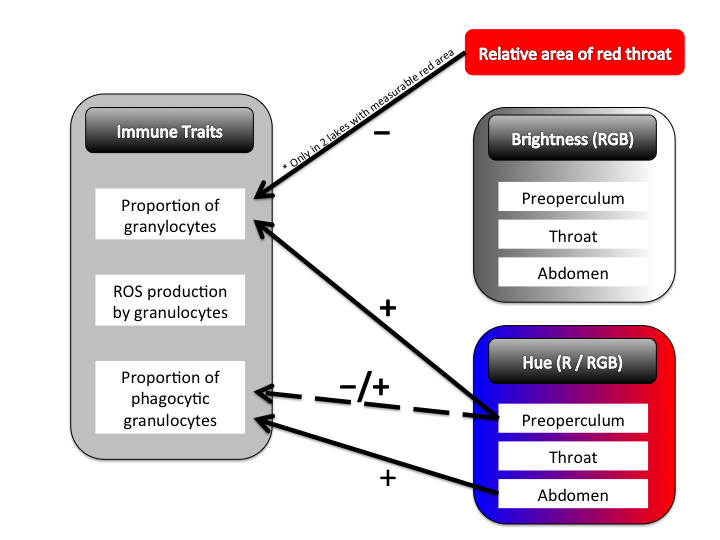


**Figure N in S2 File.** Summary of color-immune associations inferred from separate linear regressions, using photographic color measurements. Solid dark lines represent significant main effects of color on a given immune trait. Dashed lines represent significant lake by color interactions, indicating population-specific color-immune relationships. Plus or minus signs indicate effect direction. For lake*color interactions, we indicate whether the interaction occurs because of opposite effect directions (-/+) or because effects are present in some and absent in other lakes (e.g., -/0). For purposes of effect directions, redder males have higher hue scores.

**Table A in S2 File.** Canonical correlation analysis (CCA) of the multivariate correlation between three immune traits and multiple dimensions of male color measured via spectrophotometry. CCA was run first for all four populations combined, after first mean-standardizing each trait within each population, to avoid correlations arising from between-population differences. CCA was then run separately for each lake. For each CCA, we present the correlation coefficient (r), and an asymptotic approximation to an F statistic and P-value, obtained via the p.asym command in the R package CCP. We also present the loadings of each trait on the canonical x axis (for color) and y axis (for immune traits).

|  | All 4 lakes | Blackwater | Farewell | Gosling | Lower Stella |
| --- | --- | --- | --- | --- | --- |
| **Canonical correlation analysis** |  |  |  |  |  |
| r | 0.338 | 0.701 | 0.517 | 0.741 | 0.782 |
| P | 0.4110 | **0.0743** | 0.9540 | **0.0002** | **< 0.0001** |
|  |  |  |  |  |  |
| **Canonial X axis loadings** |  |  |  |  |  |
| Relative area of red throat | 1.62 | 0 | 0 | -2.01 | 2.19 |
| Total (R + G + B) |  |  |  |  |  |
| Abdomen | 0 | 0 | 0 | 0 | 0 |
| Preoperculum | 0 | 0 | 0 | 0 | 0 |
| Throat | 0 | 0 | 0 | 0 | 0 |
| Proportion of red (R / (R + G + B)) |  |  |  |  |  |
| Abdomen | 1.15 | -5.34 | -5.28 | 0.71 | 1.48 |
| Preoperculum | -2.90 | 6.67 | -12.84 | 10.07 | -4.99 |
| Throat | -2.87 | 9.21 | 14.82 | -3.55 | -3.86 |
|  |  |  |  |  |  |
| **Canonical Y axis loadings** |  |  |  |  |  |
| % granulocytes | 0.42 | 1 | 0.312 | 2.61 | 1.51 |
| Median ROS production | 0.121 | -0.29 | 0.186 | 0.12 | 0.06 |
| % phagocytic granulocytes | -0.08 | -0.74 | -1.27 | 0.27 | -0.23 |
