# Supplementary material for: Population-Specific Covariation between Immune Function and Color of Nesting Male Threespine Stickleback
Source: PLoS One. 2015 Jun 3;10(6):e0126000. doi: 10.1371/journal.pone.0126000 (PMC4454680; doi:10.1371/journal.pone.0126000)
Supplement: S3 File — This includes 9 pages of text, and Figs A—K, and Tables A-C. (DOCX) [file pone.0126000.s010.docx]

**S3 File**

**Supplementary results concerning**

**potential confounding variables affecting color or immunity**

Although we cannot exhaustively examine all possible confounding variables that might create spurious correlations between color and immune function, we were able to identify a number of additional factors that explain some variation in color or immune function. We examined whether immune function or color were, separately, correlated with male parasite infection status, courtship activity, parental status, date or time of capture, holding time, or size. Parasite load is a potentially important covariate that is likely to affect both male color and immune traits. Indeed, variation in parasitism of wild fish may be an essential mechanism that generates the associations between color and immunity that we here evaluate. Nesting status (courting, guarding eggs or fry) has been proposed to affect male color due to changes in hormonal concentrations, and so might be a source of variation for our study. Capture time and date might be significant effects if immunity or color changed directionally as the breeding season progressed or through diel cycles. Finally, the handling time between capture and measurement might introduce stress that might rapidly and dynamically alter both color or immune function.

We found numerous effects of covariates on immunity or color (detailed below, and graphically summarized in S5 and S6 Figures), including effects of parasite load, sample date, handling time, time of day when captured, and courtship activity. Often these effects were population-specific. These associations are described in detail in the supplemental text below . However, relatively few of these potential covariates were significantly correlated with both male color and immunity traits that were, themselves, interrelated. Consequently, few of these covariates could plausibly generate spurious correlations between immune function and color. For the few parameters with significant effects on both focal traits, we included them as covariates in the immune-color linear models describe in the main text. In all cases, color-immunity relationships remain significant even in multiple regressions that include the potentially confounding additional variables. Consequently, we opted to relegate all analyses of covariates to this supplement, because they had negligible effects on the core topic of the main text.

*Male behavior and breeding status*

Males’ courtship behavior explains some of the variation in male color. In Blackwater Lake (a predominantly melanic-male lake), the 4 males observed actively courting showed a marginal tendency to have more UV & blue abdomens than the 36 non-courting males (P = 0.079). In Gosling Lake (a predominantly red-male lake) the 5 courting males tended to have redder abdomens than the 32 non-courting males (P = 0.073). As a result, there was a significant lake by courtship interaction (P = 0.018) but no overall color effect (P = 0.75). However, courtship had no significant effect on any immune parameter within or across lakes, as main effects or interactions. An important caveat is that all males were observed for only a brief time (typically 10-15 minutes), so we almost certainly underestimated the number of actively courting males. Overall about half of the males sampled had eggs or fry in their nest. Contrary to our expectations, there was no significant association between color or immune traits and the presence/absence of offspring or their developmental stage (all P > 0.05). Other behaviors (nest building, aggressive interactions with other males, aggression against nest predators) were noted too rarely or inconsistently to provide sufficient power to justify analyses.

*Sampling date*

Collection date was the only methodological variable we found to be correlated with both color and immunity. We used linear regression to compare color or immune traits with Julian date (days since January 1) when each male was collected. Male color changed over nearly a week period within each lake. For logistical reasons, lakes were sampled in series rather than in parallel. As a result we cannot completely disentangle the long-term effects of date from effects of lake. Males in Gosling lake tended to develop more blue abdomens (proportion reflectance in the UV-blue third of the spectrum) over the 5 days they were sampled (P = 0.013), whereas other lakes showed no such trend, resulting in a lake*date interaction (Figure A in S3 File, P = 0.0056). For the throat, males of all three lakes exhibited a non-significant tendency to get redder as the season progressed. Because the trend was so similar in all three lakes, a linear model with lake, date, and lake*date effects infers a significant main effect of date (P = 0.002, Figure B in S3 File) even though the trend is not significant within each lake alone. Finally, the lower eye tended to get less blue over the week when we sampled Lower Stella Lake (P = 0.0002), but not in the other lakes, resulting in a significant lake*date interaction. No other color traits varied with date.

Turning next to immune function, we found increasing phagocytosis rates over a four-day period in Farewell Lake (P = 0.016), and a marginally significant trend in the other direction in Gosling lake (P = 0.063), resulting in a lake*date interaction (P = 0.0025; Figure C in S3 File). Other immune traits did not exhibit significant effects of date. No other immune traits varied with date. The above effects of sampling date are surprising, given that each lake was sampled within a single week, whereas the breeding season in these lakes typically lasts at least a month, from late May through early to mid July (Bolnick, pers. obs.). The cause of these short-term trends in color and immunity is unclear. It is not attributable to changes in breeding status (e.g., tending eggs or fry), as these did not change over this short time scale. It is possible that short-term changes in weather (irradiance, temperature) or prey availability are the root cause.

Given the among-day trends in male color and immunity, we worried that color-immunity relationship might be a spurious effect of their joint dependence on date. As noted in the main text, including collection date (and date*lake interaction) as covariates in our immune-color regressions does not appreciably effect our inferences regarding color-immune relationships. We found a significant effect of male color (F_1,96_ = 6.05, P = 0.0156) on phagocytosis rates, along with a marginally significant lake by date interaction (F_2,96_ = 3.06, P = 0.052). We conclude that this color-immunity association is robust to date effects.

*Time of sampling*

Male color and immune function might also jointly covary on a diel cycle. Indeed, we found a moderate tendency for Lower Stella males’ preoperculum to get redder as the capture time approached midday (Figure D in S3 File; P = 0.017 in Lower Stella alone, lake*time interaction P = 0.01 in a whole-dataset model). Note that almost all samples were done before 1 PM to ensure sufficient time for flow cytometry the same day. Also in Lower Stella, phagocytosis rates increased as the day progressed (P = 0.022) and perhaps decreased in Gosling Lake (P =0.067) resulting in a lake*time interaction (P = 0.033). However, preoperculum color and phagocytosis rates were not correlated in a univariate analysis, so inclusion of collection time had no impact on the observed color-immune associations. The relationship between phagocytosis rate and color of other body parts were unaffected by collection time. For example, abdomen color was correlated with phagocytosis rates (main effect P = 0.012; lake*color interaction P = 0.022) whether or not capture time was included in the model as a covariate (lake*time P = 0.014). The model presented in the main text, comparing abdomen color to phagocytosis rates, includes both collection time and collection date as covariates (with time and date interactions with lake), and abdomen color continues to have a significant association with phagocytosis.

*Handling time*

Fish color may be highly plastic to changes in light environment, social interactions, or handling stress. We tried to mitigate light and social effects by holding fish individually in dark cages, but handling effects were inevitable. To assay such handling effects we tested whether color or immunity covary with the lag time between capture and measurement. This lag time showed significant effects on male color. Fish from both Gosling and Lower Stella lakes showed a significant tendency to lose abdomen red coloration (become proportionally more UV & blue) the longer they were held before spec measurements (Figure E in S3 File Blackwater lake fish showed no such trend. As a result there is a lake*handling time interaction (P = 0.001). Throat color also became less red, although this trend was only significant in Lower Stella lake (P = 0.037; Figure F in S3 File). Phagocytosis rate was affected by handling time, albeit only in Gosling Lake (P = 0.039, Figure G in S3 File). Handling time ceased to have any significant effect on phagocytosis rates when added to a larger model with color, date, and lake effects, and did not affect the relationship between abdomen color and phagocytosis rates. When we re-evaluated the relationship between immune measures and male color including handling time as a covariate, most of the handling time effects ceased to be significant, and all of the color-immunity relationships described in the main text of this paper remained significant. We conclude that although holding fish for 2 to 3 hours does affect their color, those changes were not sufficient to generate spurious (or obscure real) color-immunity relationships.

*Fish mass*

Larger fish were likely to yield correspondingly larger head kidneys and therefore more cells in culture (Table A in S3 File). Indeed, the total number of viable cells (granulocytes + lymphocytes) recorded by flow cytometry was positively related to fish mass (P = 0.0012). Lakes differed in the number of cells per gram body mass (lake effect P < 0.0001). For example, Blackwater and Farewell fish are not significantly different in body mass (P = 0.71), but the former have on average 78% more cells in head kidney extracts. The allometry (slope) was similar across lakes (lake*mass interaction P = 0.743). However, the ratio of granulocytes to lymphocytes was unaffected by fish mass (P = 0.791) or the number of cells (P = 0.754). Both phagocytosis rates and ROS production by granulocytes were insensitive to the number of cells or body mass (all P > 0.05). None of the color-immune comparisons were altered when we included fish size as a covariate in the linear models.

*Parasite infections*

Although the mechanistic basis of these color-immune relationships remains uncertain, one possibility is that both immune and color traits respond to parasite infection. If so, we expect to see (1) correlations between parasite infection metrics and both color and immunity, and (2) reduced color-immune correlations when infection is included as a covariate. Therefore, after our field season when fish were collected, preserved carcasses were dissected under a stereomicroscope to enumerate and identify macroparasites to the lowest feasible taxonomic level. We examined the outer skin, gills, fins, intestines, body cavity, and eyes carefully, noting the presence and abundance of each parasite species. We considered the following parasite variables. First, we calculated the total number of macroparasite taxa observed per fish (richness). Second we calculated a measure of infection severity (relative parasite load, *RL*), calculated for each fish *i* by summing normalized parasite loads across all parasite species:

where *l_ij_* is the number of individuals of parasite species *j* in host individual *i.* Finally, we tested for effects of each common parasites that infected between 20% to 80% of hosts within at least one population (parasite prevalences summarized in Table B in S3 File). Parasites outside this range of prevalences provide insufficient variance for an effective regression analysis, given our sample sizes.

We then ran linear regression models to test for effects of parasitism (abundance of each taxa, richness, and relative load) on each male trait (brightness, color, and immune measures), including lake and lake by parasite interaction effects. We found multiple associations between infection load of single parasite taxa (which varies among lakes, Table B in S3 File), and both color and immune traits (Table C in S3 File; Figures H-K in S3 File). To give a few notable examples, fish with fewer Unionidae mollusc larvae on their gills tended to have brighter eyes (in Gosling and Lower Stella but not Blackwater lakes), brighter abdomens (significant main effect, P = 0.036), as well as redder throat and preoperculum. However, Unionidae infection had no detectable effects on any immune parameters.

Thersitina crustaceans infecting gills had lake-specific effects on lower eye, preoperculum, and throat color (more infected males tended to be redder for each body part, particularly in Gosling and Lower Stella lakes). Thersitina infection also coincided with higher ROS production in Lower Stella lake. Likewise, Proteocephalus infections were correlated with both ROS production and brightness (preoperculum and abdomen). However, because ROS and brightness or color were unrelated, Thersitina and Proteocephalus infections played no role in generating an immune-color association (non-significant whether or not these infection covariates were included in the linear regression models). In some cases, including a parasite in the color-immune regression actually strengthened the color-immune association, by explaining residual variation in the dependent variable. For instance abdomen brightness and phagocytosis rates were significant correlated (P = 0.0002); including *Diplostomum* infection intensity strengthened the effect of color (color effect P = 0.00007, parasite effect P = 0.054)

To summarize, infection loads by individual parasite taxa were often correlated with male color, and in a few cases were correlated with immune measurements. About half of these significant associations represent main effects (shared across lakes; Figures H and I in S3 File) and half entail lake*parasite interactions (lake-specific effects, Figures J and K in S3 File). Finally, both parasite species richness and total infection intensity (normalized for each parasite) were weakly correlated with male color (preoperculum hue) and phagocytosis rates. However, because these immune traits are unrelated, richness and infection intensity were not responsible for any color-immune correlation. In all cases, color-immune relationships were robust to inclusion of any parasite metric. For example, the relationship between throat color and granulocyte frequency (P = 0.0056) remained equivalently significant whether we included Unionidae infection, *Crepidostomum* infection, or parasite richness (granulocyte effect on color, P = 0.0053, 0.0044, or 0.0049 respectively; each infection metric was also correlated with throat color, P = 0.0359, P = 0.0390, P = 0.0177 respectively). Parasite effects are summarized in a graphic in S5 Figure. The fact that color-immune relationships persist whether or not we account for parasite infection suggests that variation in infection rate is not responsible for the covariation between male signals and immune phenotypes.

**Figure A in S3 File. Change in male abdomen color across Julian calendar date in three lakes**. Color is measured here as the ratio of UV and blue reflectance, to the reflectance in orange-red wavelengths. Color data was lost from Farewell Lake (which was sampled first). Linear regression trends within lake are indicated with lines, dashed if non-significant or solid if significant (in which case a P value is provided, color matched to the relevant lake data). Sample-wide statistical effects of date and lake and provided at the top right. Symbols and colors as in S2 Figure (black squares = Blackwater; green triangles = Gosling; blue diamonds = Lower Stella).

**Figure B in S3 File.** **Change in male throat color across Julian calendar date in three lakes.** Color is measured here as the ratio of UV-blue to orange-red reflectance. Linear regression trends within lake are indicated with dashed lines (none are significant). Sample-wide statistical effects of date and lake and provided at the top left. Because we first account for effects of lake, the inferred main effect of date is for males to become redder (lower UV:orange ratio) with date, an effect which is shared by all three lakes. This is noteworthy because males in these three lakes exhibit very different overall coloration, particularly blackwater lake (black dots) which is very blue-black and has no red discernable to the human eye. Symbols and colors as in S2 Figure (black squares = Blackwater; green triangles = Gosling; blue diamonds = Lower Stella).

**Figure C in S3 File. Change in immune function (phagocytosis) across Julian calendar date in four lakes.** Symbols and colors as in S2 Figure (black squares = Blackwater; red circles = Farewell; green triangles = Gosling; blue diamonds = Lower Stella). Linear regression trends within lake are indicated with lines, dashed if non-significant or solid if significant (with correspondingly colored P-value). Sample-wide statistical effects of date and lake and provided at the top right.

**Figure D in S3 File. Change in male preoperculum color as a function of capture time**. Color is here measured as the ratio of UV-blue to orange-red reflectance. Symbols and colors as in S2 Figure (black squares = Blackwater; green triangles = Gosling; blue diamonds = Lower Stella). Linear regression trends within lake are indicated with lines, dashed if non-significant or solid if significant (with correspondingly colored P-value). Sample-wide statistical effects of date and lake and provided at the top right.

**Figure E in S3 File. Change in male abdomen color as a function of the lag time between capture and measurement.** Color is here measured as the ratio of UV-blue to orange-red reflectance. Symbols and colors as in S2 Figure (black squares = Blackwater; green triangles = Gosling; blue diamonds = Lower Stella). Linear regression trends within lake are indicated with lines, dashed if non-significant or solid if significant (with correspondingly colored P-value). Sample-wide statistical effects of date and lake and provided at the top left.

**Figure F in S3 File. Change in male throat color as a function of the lag time between capture and measurement.** Color is here measured as the ratio of UV-blue to orange-red reflectance. Symbols and colors as in S2 Figure (black squares = Blackwater; green triangles = Gosling; blue diamonds = Lower Stella). Linear regression trends within lake are indicated with lines, dashed if non-significant or solid if significant (with correspondingly colored P-value). Sample-wide statistical effects of date and lake and provided at the top left.

**Figure G in S3 File. Change in phagocytosis rates as a function of the lag time between capture and measurement.** Symbols and colors as in S2 Figure (black squares = Blackwater; red circles = Farewell; green triangles = Gosling; blue diamonds = Lower Stella). Linear regression trends within lake are indicated with lines, dashed if non-significant or solid if significant (with correspondingly colored P-value). Sample-wide statistical effects of date and lake and provided at the bottom left.

**Figure H in S3 File. Fish with generally more intense infections (averaging across parasites) exhibit lower phagocytosis activity by granulocytes.** The trend approaches significance in both Blackwater and Gosling Lakes (P <0.06), and is in the same direction in Lower Stella, resulting in a significant main effect of parasite load (P = 0.010, Table C in S3 File). Symbols and colors as in S2 Figure (black squares = Blackwater; green triangles = Gosling; blue diamonds = Lower Stella). Thin dashed lines are non-significant, bold dashed lines are marginally significant (0.05 < P < 0.06).

**Figure I in S3 File. Fish with more intense Proteocephalus infections exhibit higher ROS production by granulocytes, following PMA stimulation.** The trend is significant in both lakes where Proteocephalus is found, but appears to be steeper in Blackwater Lake resulting in both a main effect of infection and a Lake*infection interaction (P = 0.028, P = 0.0003 respectively, Table S7). Symbols and colors as in S2 Figure (black squares = Blackwater; green triangles = Gosling; blue diamonds = Lower Stella).

**Figure J in S3 File. Fish with more intense Unionidae infections on their gills have darker (less bright) eyes.** The trend is significant in Gosling and Lower Stella Lakes, and non-significant in Blackwater, resulting in a significant infection*Lake interaction (Table S7) and no significant main effect. Symbols and colors as in S2 Figure (black squares = Blackwater; green triangles = Gosling; blue diamonds = Lower Stella). Solid lines are significant, dashed lines non-significant.

**Figure K in S3 File. Fish with generally more intense infections (averaging across parasites) have redder (less blue) preoperculum in Gosling Lake, but tend (non-significantly) in the opposite direction in the other two lakes.** The result is a significant interaction between infection status and lake (P = 0.0045, Table S7). Symbols and colors as in S2 Figure (black squares = Blackwater; green triangles = Gosling; blue diamonds = Lower Stella). Solid lines are significant, dashed lines non-significant.

**Table A in S3 File**. Correlations among body mass and immune variables. Numbers above the diagonal are correlation coefficients between variables that have been mean-standardized by their lake mean to avoid having between-lake differences generate correlations between traits. Asterisks indicate statistical significance (‘ P < 0.1, * P < 0.05, *** P < 0.001). Below the diagonal are p-values testing the null hypothesis that correlations are similar in all lakes (e.g., significant effects indicate that the correlation varies among lakes).

|  | Mass | Cell count | % granulocytes | ROS burst | Phagocytosis |
| --- | --- | --- | --- | --- | --- |
| Mass |  | 0.327 *** | -0.003 | 0.075 | -0.173 ‘ |
| Cell count | 0.020 * |  | -0.031 | 0.204 * | -0.162 |
| % Granulocytes | 0.851 | 0.907 |  | -0.015 | -0.071 |
| ROS burst | 0.982 | 0.067 | 0.872 |  | -0.087 |
| Phagocytosis | 0.256 | 0.050 | 0.266 | 0.327 |  |

**Table B in S3 File.** Parasite community composition in the three lakes with spectrophotometric and immune data. We provide data on the parasites observed infecting at least 10% of fish in at least one lake. For each of the three lakes examined, we present the number of fish examined for parasites (sample size). Then, for each parasite we show two rows of information. The top row shows the prevalence of each parasite for each population (the percentage of fish infected by that parasite). The bottom row shows the the infection intensity, measured as the mean number of parasites per infected individual. The rightmost columns indicate whether parasite prevalence differs significantly between the populations (χ^2^. For analyses of infection-color and infection-immune trait correlations, we focus on parasites infecting at least 20% of fish in at least one lake; lower prevalences confer insufficient statistical power to be worth the additional statistical comparisons.

|  |  | Blackwater | Farewell | Gosling | Lower Stella | Test statistic | P |
| --- | --- | --- | --- | --- | --- | --- | --- |
| Sample size |  | 39 | 28 | 45 | 47 |  |  |
| Apatemon | Prevalence  Intensity | 10.3 %  1.8 | 35.7%  2.0 | 11.1 %  1.4 | 6.4 %  1.3 | χ^2 =^ 14.1  F_3,18_ = 0.2 | 0.003  0.893 |
| Bunoderina | Prevalence  Intensity | 5.1 %  1.5 | 7.1%  2.0 | 15.6 %  1.4 | 10.6 %  1.4 | χ^2 =^ 2.8  F_3,12_ = 0.2 | 0.418  0.883 |
| Crepisdopodium | Prevalence  Intensity | 43.6 %  2.2 | 50 %  2.7 | 37.8 %  2.0 | 34 %  1.8 | χ^2 =^ 2.2  F_3,60_ = 0.2 | 0.541  0.913 |
| Diplostomum | Prevalence  Intensity | 38.5 %  5.2 | 82.1 %  5.4 | 33.3 %  2.1 | 93.6 %  16.2 | χ^2 =^ 49.1  F_3,93_ = 29.8 | <0.001  <0.001 |
| Eustrongyloides | Prevalence  Intensity | 17.9 %  1.3 | 3.6 %  1.0 | 8.9 %  1.0 | 6.4 %  1.3 | χ^2 =^ 5.0  F_3,11_ = 0.3 | 0.175  0.81 |
| Neoechinorhynchus | Prevalence  Intensity | 10.3 %  1.3 | 14.3 %  1.3 | 6.7 %  1.7 | 0 %  - | χ^2 =^ 6.5  F_2,8_ = 0.2 | 0.088  0.813 |
| Proteocephalus | Prevalence  Intensity | 2.6 %  1.0 | 3.6 %  2.0 | 66.7 %  8.0 | 0 %  - | χ^2 =^ 84.7  F_2,29_ = 0.9 | <0.001  0.434 |
| Schistocephalus | Prevalence  Intensity | 0 %  - | 0 %  - | 13.3 %  1.0 | 0 %  - | χ^2 =^ 15.8  - | 0.001  - |
| Thersitina (on gills) | Prevalence  Intensity | 100 %  13.2 | 78.6 %  4.3 | 2.2 %  4.0 | 74.5 %  4.3 | χ^2 =^ 97.5  F_3,93_ = 14.7 | <0.001  <0.001 |
| Unionidae (external) | Prevalence  Intensity | 12.8 %  1.4 | 28.6 %  2.0 | 24.4 %  2.5 | 21.3 %  1.7 | χ^2 =^ 2.8  F_3,30_ = 0.2 | 0.421  0.898 |
| Unionidae (on gills) | Prevalence  Intensity | 94.9 %  8.9 | 100%  23.2 | 82.2 %  10.1 | 87.2 %  4.1 | χ^2 =^ 7.52  F_3,139_ = 25.4 | 0.057  < 0.001 |
| Unknown cestode | Prevalence  Intensity | 0 %  - | 0%  - | 26.7 %  2.2 | 0 %  - | χ^2 =^ 15.8  - | 0.001  - |
| Species richness mean (se) |  | 3.36 (0.18) | 4.03 (0.23) | 3.47 (0.23) | 3.34 (0.11) | F_3,155_ = 2.45 | 0.064 |

**Table C in S3 File**. Results of linear models testing for associations between measures of parasite load and either host immune phenotypes or host color traits. Parasite metrics include the abundance of the nine most common taxa, total species richness per fish, and relative load (normalized by maximum observed load, and summed across parasite taxa). For each combination of parasite load and host trait, we report summary statistics for effect sizes including lake effects, parasite variable effects, and lake by parasite interaction effects. We also provide the correlation coefficient for each parasite-trait combination within each of three focal lakes (Farewell is omitted due to the loss of spectrometer data). Asterisks denote statistically significant effects (* P < 0.05, ** P < 0.01, *** P < 0.001) prior to correcting for multiple comparisons. After applying sequential Bonferroni corrections, only effects marked with three asterisks (***) remain significant.

|  |  | **Lake effect** | |  | **Parasite effect** | | |  | **Lake * Parasite effect** | | | | **Correlation coefficients within lakes** | | | | |  |
| --- | --- | --- | --- | --- | --- | --- | --- | --- | --- | --- | --- | --- | --- | --- | --- | --- | --- | --- |
| **Dependent variable** | **Parasite variable** | **F** | **df** | **P** | **F** | **df** | **P** |  | **F** | **df** | **P** |  | **Blackwater** |  | **Gosling** |  | **Lower Stella** |  |
| Proportion of granulocytes | Bunoderina | 4.86 | 2 | 0.0095 | 0.02 | 1 | 0.8790 |  | 1.49 | 2 | 0.2304 |  | 0.198 |  | -0.193 |  | 0.096 |  |
| Proportion of granulocytes | Eustrongyloides | 4.80 | 2 | 0.0100 | 0.16 | 1 | 0.6891 |  | 0.77 | 2 | 0.4671 |  | 0.068 |  | -0.103 |  | -0.164 |  |
| Proportion of granulocytes | Thersitina | 4.84 | 2 | 0.0096 | 1.40 | 1 | 0.2387 |  | 0.63 | 2 | 0.5327 |  | -0.225 |  | 0.132 |  | 0.028 |  |
| Proportion of granulocytes | Unionidae (external) | 4.76 | 2 | 0.0104 | 0.45 | 1 | 0.5040 |  | 0.07 | 2 | 0.9369 |  | 0.020 |  | 0.127 |  | 0.068 |  |
| Proportion of granulocytes | Unionidae (on gills) | 4.73 | 2 | 0.0106 | 0.01 | 1 | 0.9028 |  | 0.02 | 2 | 0.9763 |  | -0.033 |  | -0.007 |  | 0.018 |  |
| Proportion of granulocytes | Proteocephalus | 4.03 | 1 | 0.0484 | 0.30 | 1 | 0.5846 |  | 0.75 | 1 | 0.3886 |  | 0.124 |  | 0.107 |  | NA |  |
| Proportion of granulocytes | Crepisdopodium | 4.99 | 2 | 0.0084 | 3.12 | 1 | 0.0800 |  | 1.45 | 2 | 0.2398 |  | -0.011 |  | -0.405 | ** | -0.217 |  |
| Proportion of granulocytes | Diplostomum | 4.76 | 2 | 0.0104 | 0.00 | 1 | 0.9529 |  | 0.38 | 2 | 0.6861 |  | -0.097 |  | 0.118 |  | -0.031 |  |
| Proportion of granulocytes | Apatemon | 4.79 | 2 | 0.0101 | 1.12 | 1 | 0.2928 |  | 0.10 | 2 | 0.9086 |  | 0.085 |  | 0.102 |  | 0.128 |  |
| Proportion of granulocytes | Species richness | 4.74 | 2 | 0.0106 | 0.04 | 1 | 0.8502 |  | 0.11 | 2 | 0.8921 |  | 0.027 |  | 0.070 |  | -0.052 |  |
| Proportion of granulocytes | Relative parasite load | 4.75 | 2 | 0.0105 | 0.03 | 1 | 0.8694 |  | 0.19 | 2 | 0.8295 |  | -0.086 |  | 0.054 |  | -0.021 |  |
| ROS production | Bunoderina | 14.72 | 2 | 0.0000 | 1.76 | 1 | 0.1877 |  | 0.48 | 2 | 0.6186 |  | 0.209 |  | 0.103 |  | 0.125 |  |
| ROS production | Eustrongyloides | 14.41 | 2 | 0.0000 | 0.00 | 1 | 0.9855 |  | 0.21 | 2 | 0.8133 |  | -0.052 |  | 0.051 |  | 0.073 |  |
| ROS production | Thersitina | 15.28 | 2 | 0.0000 | 0.02 | 1 | 0.8840 |  | 3.43 | 2 | 0.0360 | * | -0.154 |  | -0.052 |  | 0.342 | * |
| ROS production | Unionidae (external) | 14.43 | 2 | 0.0000 | 0.37 | 1 | 0.5466 |  | 0.10 | 2 | 0.9012 |  | 0.106 |  | 0.007 |  | 0.052 |  |
| ROS production | Unionidae (on gills) | 14.69 | 2 | 0.0000 | 0.74 | 1 | 0.3932 |  | 0.87 | 2 | 0.4237 |  | -0.154 |  | -0.188 |  | 0.126 |  |
| ROS production | Proteocephalus | 3.41 | 1 | 0.0691 | 5.06 | 1 | 0.0275 | * | 14.47 | 1 | 0.0003 | *** | 0.498 | ** | 0.386 | * | NA |  |
| ROS production | Crepisdopodium | 14.59 | 2 | 0.0000 | 0.54 | 1 | 0.4656 |  | 0.61 | 2 | 0.5472 |  | -0.194 |  | -0.018 |  | 0.072 |  |
| ROS production | Diplostomum | 14.83 | 2 | 0.0000 | 1.64 | 1 | 0.2031 |  | 0.92 | 2 | 0.4001 |  | -0.118 |  | 0.079 |  | -0.240 |  |
| ROS production | Apatemon | 14.39 | 2 | 0.0000 | 0.15 | 1 | 0.7020 |  | 0.03 | 2 | 0.9702 |  | 0.068 |  | 0.035 |  | -0.003 |  |
| ROS production | Species richness | 14.62 | 2 | 0.0000 | 0.05 | 1 | 0.8190 |  | 0.96 | 2 | 0.3866 |  | -0.128 |  | 0.067 |  | 0.160 |  |
| ROS production | Relative parasite load | 14.45 | 2 | 0.0000 | 0.34 | 1 | 0.5600 |  | 0.16 | 2 | 0.8504 |  | -0.035 |  | -0.033 |  | -0.113 |  |
| Phagocytosis rate | Bunoderina | 32.08 | 2 | 0.0000 | 3.68 | 1 | 0.0579 |  | 0.43 | 2 | 0.6518 |  | -0.127 |  | -0.229 |  | -0.033 |  |
| Phagocytosis rate | Eustrongyloides | 31.01 | 2 | 0.0000 | 0.84 | 1 | 0.3629 |  | 0.05 | 2 | 0.9540 |  | -0.224 |  | -0.046 |  | 0.021 |  |
| Phagocytosis rate | Thersitina | 31.80 | 2 | 0.0000 | 0.20 | 1 | 0.6558 |  | 1.71 | 2 | 0.1865 |  | -0.068 |  | 0.205 |  | 0.118 |  |
| Phagocytosis rate | Unionidae (external) | 31.04 | 2 | 0.0000 | 0.34 | 1 | 0.5620 |  | 0.35 | 2 | 0.7038 |  | -0.168 |  | 0.069 |  | 0.147 |  |
| Phagocytosis rate | Unionidae (on gills) | 31.74 | 2 | 0.0000 | 0.03 | 1 | 0.8559 |  | 1.68 | 2 | 0.1911 |  | -0.385 | * | 0.011 |  | 0.309 |  |
| Phagocytosis rate | Proteocephalus | 0.50 | 1 | 0.4815 | 2.17 | 1 | 0.1449 |  | 0.07 | 1 | 0.7927 |  | 0.068 |  | -0.183 |  | NA |  |
| Phagocytosis rate | Crepisdopodium | 31.77 | 2 | 0.0000 | 0.11 | 1 | 0.7352 |  | 1.69 | 2 | 0.1892 |  | 0.319 |  | -0.139 |  | -0.179 |  |
| Phagocytosis rate | Diplostomum | 32.98 | 2 | 0.0000 | 3.06 | 1 | 0.0834 |  | 2.26 | 2 | 0.1090 |  | -0.073 |  | 0.299 | * | 0.032 |  |
| Phagocytosis rate | Apatemon | 31.86 | 2 | 0.0000 | 1.70 | 1 | 0.1952 |  | 1.06 | 2 | 0.3506 |  | -0.482 | ** | 0.035 |  | -0.106 |  |
| Phagocytosis rate | Species richness | 30.99 | 2 | 0.0000 | 0.31 | 1 | 0.5775 |  | 0.28 | 2 | 0.7574 |  | -0.077 |  | 0.063 |  | 0.203 |  |
| Phagocytosis rate | Relative parasite load | 32.81 | 2 | 0.0000 | 6.83 | 1 | 0.0103 | * | 0.09 | 2 | 0.9098 |  | -0.337 |  | -0.243 |  | -0.158 |  |
| Lower eye brightness | Bunoderina | 2.37 | 2 | 0.0976 | 0.00 | 1 | 0.9937 |  | 0.14 | 2 | 0.8723 |  | -0.037 |  | -0.033 |  | 0.062 |  |
| Lower eye brightness | Eustrongyloides | 2.43 | 2 | 0.0921 | 1.04 | 1 | 0.3093 |  | 1.10 | 2 | 0.3373 |  | -0.190 |  | 0.249 |  | -0.083 |  |
| Lower eye brightness | Thersitina | 2.44 | 2 | 0.0918 | 2.30 | 1 | 0.1323 |  | 0.54 | 2 | 0.5839 |  | -0.213 |  | 0.157 |  | -0.107 |  |
| Lower eye brightness | Unionidae (external) | 2.38 | 2 | 0.0972 | 0.29 | 1 | 0.5908 |  | 0.09 | 2 | 0.9162 |  | 0.070 |  | -0.008 |  | 0.066 |  |
| Lower eye brightness | Unionidae (on gills) | 2.54 | 2 | 0.0835 | 0.51 | 1 | 0.4759 |  | 3.88 | 2 | 0.0235 | * | 0.174 |  | -0.291 |  | -0.315 | * |
| Lower eye brightness | Proteocephalus | 0.21 | 1 | 0.6481 | 0.07 | 1 | 0.7982 |  | 0.26 | 1 | 0.6119 |  | -0.066 |  | -0.059 |  | NA |  |
| Lower eye brightness | Crepisdopodium | 2.47 | 2 | 0.0893 | 0.00 | 1 | 0.9901 |  | 2.40 | 2 | 0.0952 |  | -0.139 |  | -0.058 |  | 0.270 |  |
| Lower eye brightness | Diplostomum | 2.41 | 2 | 0.0944 | 0.03 | 1 | 0.8731 |  | 0.98 | 2 | 0.3783 |  | -0.170 |  | 0.169 |  | -0.024 |  |
| Lower eye brightness | Apatemon | 2.39 | 2 | 0.0961 | 0.79 | 1 | 0.3758 |  | 0.13 | 2 | 0.8807 |  | -0.096 |  | -0.002 |  | -0.107 |  |
| Lower eye brightness | Species richness | 2.48 | 2 | 0.0885 | 1.97 | 1 | 0.1626 |  | 1.63 | 2 | 0.2001 |  | -0.293 |  | 0.031 |  | -0.105 |  |
| Lower eye brightness | Relative parasite load | 2.38 | 2 | 0.0966 | 0.00 | 1 | 0.9882 |  | 0.39 | 2 | 0.6804 |  | -0.100 |  | 0.040 |  | 0.068 |  |
| Preoperculum brightness | Bunoderina | 19.89 | 2 | 0.0000 | 0.05 | 1 | 0.8159 |  | 0.59 | 2 | 0.5554 |  | -0.255 |  | 0.053 |  | -0.138 |  |
| Preoperculum brightness | Eustrongyloides | 21.60 | 2 | 0.0000 | 0.02 | 1 | 0.8830 |  | 5.65 | 2 | 0.0045 | ** | -0.145 |  | 0.322 | * | -0.245 |  |
| Preoperculum brightness | Thersitina | 19.93 | 2 | 0.0000 | 0.45 | 1 | 0.5018 |  | 0.49 | 2 | 0.6112 |  | -0.095 |  | -0.108 |  | -0.126 |  |
| Preoperculum brightness | Unionidae (external) | 19.69 | 2 | 0.0000 | 0.05 | 1 | 0.8172 |  | 0.01 | 2 | 0.9858 |  | 0.087 |  | 0.010 |  | 0.020 |  |
| Preoperculum brightness | Unionidae (on gills) | 19.94 | 2 | 0.0000 | 0.21 | 1 | 0.6440 |  | 0.66 | 2 | 0.5212 |  | 0.064 |  | -0.021 |  | -0.251 |  |
| Preoperculum brightness | Proteocephalus | 33.26 | 1 | 0.0000 | 11.14 | 1 | 0.0013 | ** | 0.07 | 1 | 0.7862 |  | 0.116 |  | 0.372 | * | NA |  |
| Preoperculum brightness | Crepisdopodium | 19.72 | 2 | 0.0000 | 0.07 | 1 | 0.7910 |  | 0.07 | 2 | 0.9336 |  | 0.041 |  | -0.002 |  | 0.092 |  |
| Preoperculum brightness | Diplostomum | 20.06 | 2 | 0.0000 | 1.89 | 1 | 0.1714 |  | 0.16 | 2 | 0.8532 |  | -0.279 |  | -0.111 |  | -0.142 |  |
| Preoperculum brightness | Apatemon | 19.87 | 2 | 0.0000 | 0.07 | 1 | 0.7940 |  | 0.54 | 2 | 0.5858 |  | -0.142 |  | -0.072 |  | 0.151 |  |
| Preoperculum brightness | Species richness | 20.34 | 2 | 0.0000 | 0.46 | 1 | 0.4981 |  | 1.70 | 2 | 0.1877 |  | -0.174 |  | 0.187 |  | -0.150 |  |
| Preoperculum brightness | Relative parasite load | 19.99 | 2 | 0.0000 | 0.10 | 1 | 0.7515 |  | 0.86 | 2 | 0.4266 |  | -0.179 |  | 0.125 |  | -0.094 |  |
| Throat brightness | Bunoderina | 0.54 | 2 | 0.5853 | 0.50 | 1 | 0.4810 |  | 0.06 | 2 | 0.9440 |  | 0.004 |  | 0.120 |  | 0.078 |  |
| Throat brightness | Eustrongyloides | 0.55 | 2 | 0.5761 | 0.13 | 1 | 0.7185 |  | 1.97 | 2 | 0.1434 |  | -0.108 |  | 0.364 | * | -0.110 |  |
| Throat brightness | Thersitina | 0.55 | 2 | 0.5813 | 0.19 | 1 | 0.6643 |  | 0.96 | 2 | 0.3873 |  | -0.130 |  | 0.025 |  | 0.161 |  |
| Throat brightness | Unionidae (external) | 0.55 | 2 | 0.5783 | 0.39 | 1 | 0.5359 |  | 1.42 | 2 | 0.2452 |  | -0.188 |  | 0.211 |  | -0.112 |  |
| Throat brightness | Unionidae (on gills) | 0.56 | 2 | 0.5720 | 3.66 | 1 | 0.0583 |  | 0.98 | 2 | 0.3782 |  | -0.099 |  | -0.230 |  | -0.274 |  |
| Throat brightness | Proteocephalus | 0.06 | 1 | 0.7998 | 0.71 | 1 | 0.4024 |  | 2.79 | 1 | 0.0990 |  | 0.228 |  | 0.160 |  | NA |  |
| Throat brightness | Crepisdopodium | 0.54 | 2 | 0.5827 | 0.21 | 1 | 0.6440 |  | 0.68 | 2 | 0.5093 |  | -0.072 |  | 0.164 |  | 0.122 |  |
| Throat brightness | Diplostomum | 0.55 | 2 | 0.5792 | 0.04 | 1 | 0.8390 |  | 1.43 | 2 | 0.2431 |  | -0.222 |  | 0.041 |  | 0.109 |  |
| Throat brightness | Apatemon | 0.54 | 2 | 0.5831 | 0.31 | 1 | 0.5773 |  | 0.55 | 2 | 0.5763 |  | -0.095 |  | 0.141 |  | -0.107 |  |
| Throat brightness | Species richness | 0.56 | 2 | 0.5731 | 0.01 | 1 | 0.9038 |  | 2.60 | 2 | 0.0784 |  | -0.268 |  | 0.276 |  | 0.059 |  |
| Throat brightness | Relative parasite load | 0.55 | 2 | 0.5809 | 0.00 | 1 | 0.9713 |  | 1.12 | 2 | 0.3290 |  | -0.173 |  | 0.203 |  | -0.009 |  |
| Abdomen brightness | Bunoderina | 6.68 | 2 | 0.0018 | 0.05 | 1 | 0.8318 |  | 0.17 | 2 | 0.8461 |  | -0.102 |  | -0.007 |  | 0.020 |  |
| Abdomen brightness | Eustrongyloides | 6.67 | 2 | 0.0018 | 0.19 | 1 | 0.6605 |  | 0.04 | 2 | 0.9563 |  | 0.063 |  | 0.065 |  | 0.004 |  |
| Abdomen brightness | Thersitina | 6.76 | 2 | 0.0017 | 1.63 | 1 | 0.2043 |  | 0.15 | 2 | 0.8577 |  | -0.185 |  | -0.141 |  | -0.032 |  |
| Abdomen brightness | Unionidae (external) | 6.80 | 2 | 0.0016 | 1.48 | 1 | 0.2255 |  | 0.50 | 2 | 0.6064 |  | -0.165 |  | 0.031 |  | -0.175 |  |
| Abdomen brightness | Unionidae (on gills) | 7.09 | 2 | 0.0012 | 4.49 | 1 | 0.0362 | * | 1.59 | 2 | 0.2087 |  | -0.079 |  | -0.242 |  | -0.324 | * |
| Abdomen brightness | Proteocephalus | 2.18 | 1 | 0.1441 | 6.19 | 1 | 0.0150 | * | 0.20 | 1 | 0.6592 |  | 0.074 |  | 0.388 | * | NA |  |
| Abdomen brightness | Crepisdopodium | 6.85 | 2 | 0.0015 | 1.13 | 1 | 0.2909 |  | 1.14 | 2 | 0.3249 |  | 0.071 |  | -0.005 |  | 0.256 |  |
| Abdomen brightness | Diplostomum | 6.84 | 2 | 0.0016 | 1.06 | 1 | 0.3048 |  | 1.06 | 2 | 0.3505 |  | 0.238 |  | -0.061 |  | 0.145 |  |
| Abdomen brightness | Apatemon | 6.81 | 2 | 0.0016 | 2.36 | 1 | 0.1269 |  | 0.19 | 2 | 0.8288 |  | 0.217 |  | 0.171 |  | 0.034 |  |
| Abdomen brightness | Species richness | 6.67 | 2 | 0.0018 | 0.11 | 1 | 0.7451 |  | 0.09 | 2 | 0.9103 |  | -0.071 |  | 0.011 |  | -0.049 |  |
| Abdomen brightness | Relative parasite load | 6.86 | 2 | 0.0015 | 2.79 | 1 | 0.0976 |  | 0.44 | 2 | 0.6425 |  | 0.235 |  | 0.086 |  | 0.171 |  |
| Lower eye blue:red ratio | Bunoderina | 49.19 | 2 | 0.0000 | 0.57 | 1 | 0.4521 |  | 0.25 | 2 | 0.7831 |  | 0.084 |  | -0.102 |  | -0.090 |  |
| Lower eye blue:red ratio | Eustrongyloides | 49.20 | 2 | 0.0000 | 0.00 | 1 | 0.9998 |  | 0.54 | 2 | 0.5835 |  | -0.168 |  | 0.088 |  | 0.072 |  |
| Lower eye blue:red ratio | Thersitina | 55.99 | 2 | 0.0000 | 1.29 | 1 | 0.2578 |  | 7.97 | 2 | 0.0006 | *** | 0.042 |  | -0.503 | *** | -0.161 |  |
| Lower eye blue:red ratio | Unionidae (external) | 50.46 | 2 | 0.0000 | 3.15 | 1 | 0.0788 |  | 0.48 | 2 | 0.6219 |  | 0.001 |  | 0.114 |  | 0.262 |  |
| Lower eye blue:red ratio | Unionidae (on gills) | 49.40 | 2 | 0.0000 | 0.01 | 1 | 0.9085 |  | 0.78 | 2 | 0.4609 |  | -0.120 |  | -0.052 |  | 0.159 |  |
| Lower eye blue:red ratio | Proteocephalus | 98.54 | 1 | 0.0000 | 0.32 | 1 | 0.5745 |  | 3.39 | 1 | 0.0696 |  | 0.450 | ** | -0.074 |  | NA |  |
| Lower eye blue:red ratio | Crepisdopodium | 51.24 | 2 | 0.0000 | 2.92 | 1 | 0.0902 |  | 1.51 | 2 | 0.2254 |  | 0.030 |  | 0.174 |  | 0.290 |  |
| Lower eye blue:red ratio | Diplostomum | 59.08 | 2 | 0.0000 | 11.44 | 1 | 0.0010 | *** | 6.58 | 2 | 0.0020 | ** | -0.196 |  | -0.600 | *** | -0.061 |  |
| Lower eye blue:red ratio | Apatemon | 49.90 | 2 | 0.0000 | 0.57 | 1 | 0.4531 |  | 1.09 | 2 | 0.3393 |  | -0.198 |  | 0.117 |  | -0.171 |  |
| Lower eye blue:red ratio | Species richness | 49.25 | 2 | 0.0000 | 0.36 | 1 | 0.5489 |  | 0.43 | 2 | 0.6548 |  | -0.037 |  | -0.133 |  | 0.067 |  |
| Lower eye blue:red ratio | Relative parasite load | 49.41 | 2 | 0.0000 | 1.21 | 1 | 0.2729 |  | 0.18 | 2 | 0.8323 |  | -0.233 |  | -0.121 |  | -0.007 |  |
| Preoperculum blue:red ratio | Bunoderina | 6.76 | 2 | 0.0017 | 6.71 | 1 | 0.0108 | * | 2.23 | 2 | 0.1124 |  | 0.145 |  | -0.429 | ** | -0.183 |  |
| Preoperculum blue:red ratio | Eustrongyloides | 6.43 | 2 | 0.0023 | 1.45 | 1 | 0.2304 |  | 1.72 | 2 | 0.1831 |  | 0.280 |  | -0.190 |  | 0.137 |  |
| Preoperculum blue:red ratio | Thersitina | 6.65 | 2 | 0.0018 | 2.35 | 1 | 0.1276 |  | 3.36 | 2 | 0.0382 | * | -0.076 |  | -0.259 |  | -0.353 | * |
| Preoperculum blue:red ratio | Unionidae (external) | 6.41 | 2 | 0.0023 | 2.94 | 1 | 0.0892 |  | 0.80 | 2 | 0.4498 |  | 0.163 |  | 0.277 |  | 0.064 |  |
| Preoperculum blue:red ratio | Unionidae (on gills) | 6.64 | 2 | 0.0019 | 6.38 | 1 | 0.0129 | * | 1.27 | 2 | 0.2843 |  | 0.306 |  | 0.349 | * | -0.050 |  |
| Preoperculum blue:red ratio | Proteocephalus | 0.28 | 1 | 0.5964 | 2.08 | 1 | 0.1532 |  | 0.48 | 1 | 0.4895 |  | 0.119 |  | -0.210 |  | NA |  |
| Preoperculum blue:red ratio | Crepisdopodium | 6.26 | 2 | 0.0026 | 0.95 | 1 | 0.3317 |  | 0.39 | 2 | 0.6772 |  | 0.188 |  | -0.026 |  | 0.123 |  |
| Preoperculum blue:red ratio | Diplostomum | 6.37 | 2 | 0.0024 | 0.01 | 1 | 0.9436 |  | 1.89 | 2 | 0.1562 |  | 0.184 |  | -0.227 |  | 0.094 |  |
| Preoperculum blue:red ratio | Apatemon | 6.53 | 2 | 0.0020 | 4.12 | 1 | 0.0446 | * | 1.41 | 2 | 0.2483 |  | 0.268 |  | 0.315 | * | -0.020 |  |
| Preoperculum blue:red ratio | Species richness | 6.41 | 2 | 0.0023 | 0.34 | 1 | 0.5638 |  | 2.14 | 2 | 0.1227 |  | 0.350 | * | -0.019 |  | -0.139 |  |
| Preoperculum blue:red ratio | Relative parasite load | 6.82 | 2 | 0.0016 | 0.91 | 1 | 0.3427 |  | 5.66 | 2 | 0.0045 | ** | 0.247 |  | -0.436 | ** | 0.139 |  |
| Throat blue:red ratio | Bunoderina | 47.56 | 2 | 0.0000 | 0.79 | 1 | 0.3752 |  | 0.16 | 2 | 0.8503 |  | -0.064 |  | -0.206 |  | 0.009 |  |
| Throat blue:red ratio | Eustrongyloides | 47.53 | 2 | 0.0000 | 0.76 | 1 | 0.3865 |  | 0.13 | 2 | 0.8765 |  | 0.132 |  | -0.031 |  | 0.083 |  |
| Throat blue:red ratio | Thersitina | 48.45 | 2 | 0.0000 | 0.04 | 1 | 0.8467 |  | 1.64 | 2 | 0.1994 |  | 0.147 |  | -0.138 |  | -0.185 |  |
| Throat blue:red ratio | Unionidae (external) | 49.67 | 2 | 0.0000 | 5.82 | 1 | 0.0174 | * | 0.24 | 2 | 0.7903 |  | 0.273 |  | 0.290 |  | 0.163 |  |
| Throat blue:red ratio | Unionidae (on gills) | 47.64 | 2 | 0.0000 | 0.89 | 1 | 0.3483 |  | 0.21 | 2 | 0.8133 |  | 0.047 |  | 0.141 |  | 0.112 |  |
| Throat blue:red ratio | Proteocephalus | 66.39 | 1 | 0.0000 | 0.00 | 1 | 0.9971 |  | 2.50 | 1 | 0.1183 |  | 0.231 |  | -0.004 |  | NA |  |
| Throat blue:red ratio | Crepisdopodium | 48.21 | 2 | 0.0000 | 0.99 | 1 | 0.3212 |  | 0.85 | 2 | 0.4289 |  | 0.041 |  | 0.049 |  | 0.205 |  |
| Throat blue:red ratio | Diplostomum | 49.16 | 2 | 0.0000 | 0.51 | 1 | 0.4785 |  | 2.27 | 2 | 0.1084 |  | 0.254 |  | -0.244 |  | 0.149 |  |
| Throat blue:red ratio | Apatemon | 48.75 | 2 | 0.0000 | 1.71 | 1 | 0.1940 |  | 1.17 | 2 | 0.3150 |  | 0.141 |  | 0.374 | * | -0.032 |  |
| Throat blue:red ratio | Species richness | 49.40 | 2 | 0.0000 | 4.27 | 1 | 0.0411 | * | 0.68 | 2 | 0.5079 |  | 0.355 | * | 0.168 |  | 0.099 |  |
| Throat blue:red ratio | Relative parasite load | 48.10 | 2 | 0.0000 | 0.00 | 1 | 0.9535 |  | 1.22 | 2 | 0.2998 |  | 0.039 |  | -0.218 |  | 0.144 |  |
| Abdomen blue:red ratio | Bunoderina | 7.12 | 2 | 0.0012 | 0.49 | 1 | 0.4875 |  | 0.25 | 2 | 0.7822 |  | -0.069 |  | -0.136 |  | 0.041 |  |
| Abdomen blue:red ratio | Eustrongyloides | 7.15 | 2 | 0.0012 | 1.05 | 1 | 0.3086 |  | 0.23 | 2 | 0.7973 |  | 0.199 |  | -0.039 |  | 0.083 |  |
| Abdomen blue:red ratio | Thersitina | 7.52 | 2 | 0.0009 | 0.28 | 1 | 0.5953 |  | 3.65 | 2 | 0.0291 | * | 0.281 |  | -0.242 |  | -0.231 |  |
| Abdomen blue:red ratio | Unionidae (external) | 7.12 | 2 | 0.0012 | 0.80 | 1 | 0.3740 |  | 0.09 | 2 | 0.9184 |  | 0.137 |  | 0.034 |  | 0.091 |  |
| Abdomen blue:red ratio | Unionidae (on gills) | 7.09 | 2 | 0.0012 | 0.47 | 1 | 0.4955 |  | 0.07 | 2 | 0.9296 |  | -0.037 |  | -0.097 |  | -0.062 |  |
| Abdomen blue:red ratio | Proteocephalus | 12.14 | 1 | 0.0008 | 2.22 | 1 | 0.1399 |  | 1.76 | 1 | 0.1887 |  | 0.228 |  | -0.218 |  | NA |  |
| Abdomen blue:red ratio | Crepisdopodium | 7.10 | 2 | 0.0012 | 0.12 | 1 | 0.7319 |  | 0.30 | 2 | 0.7401 |  | -0.089 |  | -0.069 |  | 0.079 |  |
| Abdomen blue:red ratio | Diplostomum | 7.11 | 2 | 0.0012 | 0.00 | 1 | 0.9972 |  | 0.44 | 2 | 0.6455 |  | -0.072 |  | -0.093 |  | 0.089 |  |
| Abdomen blue:red ratio | Apatemon | 7.19 | 2 | 0.0011 | 0.03 | 1 | 0.8549 |  | 1.06 | 2 | 0.3494 |  | 0.195 |  | -0.038 |  | -0.145 |  |
| Abdomen blue:red ratio | Species richness | 7.26 | 2 | 0.0011 | 0.00 | 1 | 0.9885 |  | 1.71 | 2 | 0.1863 |  | 0.289 |  | -0.144 |  | -0.061 |  |
| Abdomen blue:red ratio | Relative parasite load | 7.15 | 2 | 0.0012 | 0.22 | 1 | 0.6363 |  | 0.61 | 2 | 0.5439 |  | -0.071 |  | -0.140 |  | 0.101 |  |
