# Supplementary material for: Population-Specific Covariation between Immune Function and Color of Nesting Male Threespine Stickleback
Source: PLoS One. 2015 Jun 3;10(6):e0126000. doi: 10.1371/journal.pone.0126000 (PMC4454680; doi:10.1371/journal.pone.0126000)
Supplement: S1 Table — (DOCX) [file pone.0126000.s011.docx]

**Table S1.** Locations of the study populations. Two sample sizes are given for each site: the number of specimens measured by the spectrophotometer, and the number measured by flow cytometry. The numbers differ both because some spectrophotometry files were lost due to hardware failure, and because the flow cytometer failed to run some samples correctly.

| **Population** | **Watershed** | **Latitude** | **Longitude** | **Sample size (spec / flow)** |
| --- | --- | --- | --- | --- |
| Blackwater Lake | Amor de Cosmos River | 50.1780 | 125.5885 | 40 / 32 |
| Farewell Lake | Amor de Cosmos River | 50.2013 | 125.5862 | 0 / 28 |
| Gosling Lake | Campbell River | 50.0577 | 125.5028 | 45 / 50 |
| Lower Stella Lake | Pye River | 50.3118 | 125.5394 | 41 / 31 |
