# Supplementary material for: Population-Specific Covariation between Immune Function and Color of Nesting Male Threespine Stickleback
Source: PLoS One. 2015 Jun 3;10(6):e0126000. doi: 10.1371/journal.pone.0126000 (PMC4454680; doi:10.1371/journal.pone.0126000)
Supplement: S2 Table — (DOCX) [file pone.0126000.s012.docx]

**Table S2.** Results of linear models in which immune traits are tested for their association with total brightness of four male body parts, lake effects, and color by lake interaction effects. P values are presented for each effect of the linear model. For each lake, P-values for within-lake regressions are presented. Where these effects are significant or marginally significant (bold P-values), we also present effect directions (+/-) indicating whether the immune trait increases or decreases with the specified color trait.

|  |  |  |  |  | **Blackwater Lake** | | **Gosling Lake** | | **Lower Stella Lake** | |
| --- | --- | --- | --- | --- | --- | --- | --- | --- | --- | --- |
| **Body part** | **Immune trait** | **Trait effect (P)** | **Lake effect (P)** | **Trait*Lake interaction (P)** | **Effect** | **P value** | **Effect** | **P value** | **Effect** | **P value** |
| Lower eye | Proportion of granulocytes | 0.2869 | **0.0063** | 0.5364 |  | 0.9967 |  | 0.8219 |  | 0.1763 |
| Lower eye | ROS burst | 0.6379 | **< 0.0001** | 0.9886 |  | 0.7549 |  | 0.8509 |  | 0.7642 |
| Lower eye | Phagocytosis rate | 0.0542 | **< 0.0001** | 0.7346 |  | 0.1705 |  | 0.8994 | **−** | **0.0060** |
| Preoperculum | Proportion of granulocytes | 0.9585 | **0.0067** | 0.6763 |  | 0.7045 |  | 0.5650 |  | 0.5463 |
| Preoperculum | ROS burst | 0.7444 | **< 0.0001** | 0.4518 |  | 0.1953 |  | 0.9364 |  | 0.9962 |
| Preoperculum | Phagocytosis rate | 0.9859 | **< 0.0001** | **0.0303** |  | 0.2278 |  | 0.2856 | **−** | **0.0025** |
| Throat | Proportion of granulocytes | 0.7818 | **0.0069** | 0.9646 |  | 0.9736 |  | 0.7400 |  | 0.7808 |
| Throat | ROS burst | 0.8166 | **< 0.0001** | 0.8727 |  | 0.6213 |  | 0.6764 |  | 0.9802 |
| Throat | Phagocytosis rate | 0.0536 | **< 0.0001** | 0.4939 |  | 0.6409 |  | 0.2402 | **−** | **0.0243** |
| Abdomen | Proportion of granulocytes | 0.0523 | **0.0056** | 0.5361 |  | 0.1591 |  | 0.9490 |  | 0.1534 |
| Abdomen | ROS burst | 0.1150 | **< 0.0001** | 0.8805 |  | 0.3855 |  | 0.3922 |  | 0.2852 |
| Abdomen | Phagocytosis rate | **0.0003** | **< 0.0001** | 0.5188 | **−** | **0.0218** | **−** | **0.0442** | **−** | **0.0063** |
