# Supplementary material for: Population-Specific Covariation between Immune Function and Color of Nesting Male Threespine Stickleback
Source: PLoS One. 2015 Jun 3;10(6):e0126000. doi: 10.1371/journal.pone.0126000 (PMC4454680; doi:10.1371/journal.pone.0126000)
Supplement: S3 Table — (DOCX) [file pone.0126000.s013.docx]

**Table S3.** Results of linear models in which immune traits are tested for their association with color (proportion of orange-red reflectance) of four male body parts, lake effects, and color by lake interaction effects. P values are presented for each effect of the linear model. For each lake, P-values for within-lake regressions are presented. Where these effects are significant or marginally significant (bold P-values), we also present effect directions (+/-) indicating whether the immune trait increases or decreases with the specified color trait.

|  |  |  |  |  | **Blackwater Lake** | | **Gosling Lake** | | **Lower Stella Lake** | |
| --- | --- | --- | --- | --- | --- | --- | --- | --- | --- | --- |
| **Body part** | **Immune trait** | **Trait effect (P)** | **Lake effect (P)** | **Trait*Lake interaction (P)** | **Effect** | **P value** | **Effect** | **P value** | **Effect** | **P value** |
| Lower eye | Proportion of granulocytes | 0.6928 | **0.0068** | 0.7902 |  | 0.8628 |  | 0.2346 |  | 0.9923 |
| Lower eye | ROS burst | 0.1747 | **< 0.0001** | 0.7743 |  | 0.3203 |  | 0.8247 |  | 0.3391 |
| Lower eye | Phagocytosis rate | 0.2898 | **< 0.0001** | **0.0587** |  | 0.4458 | **+** | 0.0742 |  | 0.4365 |
| Preoperculum | Proportion of granulocytes | 0.1469 | **0.0059** | 0.4744 |  | 0.6005 |  | 0.8182 |  | 0.1127 |
| Preoperculum | ROS burst | 0.8299 | **< 0.0001** | 0.6886 |  | 0.9239 |  | 0.4750 |  | 0.5332 |
| Preoperculum | Phagocytosis rate | 0.8118 | **< 0.0001** | 0.7412 |  | 0.7324 |  | 0.6326 |  | 0.5762 |
| Throat | Proportion of granulocytes | **0.0025** | **0.0044** | 0.6562 |  | 0.2690 |  | 0.5800 | **−** | **0.0098** |
| Throat | ROS burst | 0.5684 | **< 0.0001** | 0.9157 |  | 0.6027 |  | 0.5749 |  | 0.8045 |
| Throat | Phagocytosis rate | 0.3445 | **< 0.0001** | 0.7673 |  | 0.9078 |  | 0.4412 |  | 0.4535 |
| Abdomen | Proportion of granulocytes | 0.8680 | **0.0063** | 0.3411 |  | 0.5061 |  | 0.8205 |  | 0.2616 |
| Abdomen | ROS burst | 0.2370 | **< 0.0001** | 0.7933 |  | 0.6201 |  | 0.5623 |  | 0.3140 |
| Abdomen | Phagocytosis rate | **0.0073** | **< 0.0001** | 0.0503 |  | 0.9523 | **+** | **0.0112** |  | 0.2562 |
